# Carbon Reduction Powered by Natural Electrochemical Gradients under Submarine Hydrothermal Vent Conditions

**DOI:** 10.1021/jacs.5c01948

**Published:** 2025-07-29

**Authors:** T. Altair, E. Dragoti, V. Sojo, Y. Li, S. Skiffington, W.A. Sullivan, G.T. Drozd, S.E. McGlynn, D. Galante, H. Varela, R. Hudson

**Affiliations:** † College of the Atlantic, Bar Harbor, Maine 04609 United States; ‡ Sao Carlos Institute of Chemistry, University of Sao Paulo, Sao Carlos 13560-970 Brazil; § Institute for Comparative Genomics & Richard Gilder Graduate School, American Museum of Natural History, New York, New York, 10024 United States; ∥ Earth-Life Science Institute, Institute of Science Tokyo, Tokyo 152-0033, Japan; ⊥ Center for Sustainable Resource Science RIKEN, Wako 351-0198 Japan; # Department of Chemistry, Colby College, Waterville, Maine 04901, United States; ∇ Department of Geology, Colby College, Waterville, Maine 04901, United States; ○ Blue Marble Space Institute of Science, Seattle, Washington 98104, United States; ◆ Institute of Geosciences, University of Sao Paulo, Sao Paulo 05508-080, Brazil

## Abstract

Energy metabolism at the emergence of life has been the topic of intense theoretical and experimental study. Alkaline hydrothermal vents (AHVs) may have facilitated energy transfer and carbon fixation at life’s emergence. Specifically, pH separation across vent walls could have been the forerunner to pH separation across cell membranes, with inorganic barriers containing [Ni-]­FeS minerals as precursors of metalloenzymes in potentially ancient biological reductive acetyl-CoA Wood–Ljungdahl (WL) and other metabolic pathways. We previously demonstrated pH-gradient-dependent reduction of CO_2_ to formate by H_2_ in AHV interface conditions. Here, we address the same problem of CO_2_ reduction using a macroscale reactor with minerals synthesized via protocols meant to mimic the natural processes of hydrothermal chimney formation. This reactor also allowed us to probe more variables and explore longer experimentation time frames. These results elucidate how different aspects of the hydrothermal–vent interface (e.g., different minerals and/or temperature gradients) affect the observed CO_2_ electrochemical reduction as well as the flow of electrons under passive vs induced currents and potentials. Using experimental simulations and electrochemistry techniques, we detected two key steps of the WL pathway (CO_2_ to formic acid and the formation of acetic acid). We explored effects of Ni incorporation in the mineral catalyst, as well as temperature and the effects of these variables on the production of formate. Currents as small as 10 nanoamps to 10 microamps were enough to efficiently carry out CO_2_ reduction. In this work, we electrochemically explore energy protometabolism in vent–ocean interfaces, specifically focusing on [Ni-]­FeS minerals as precursors of metalloenzymes.

## Graphical abstract

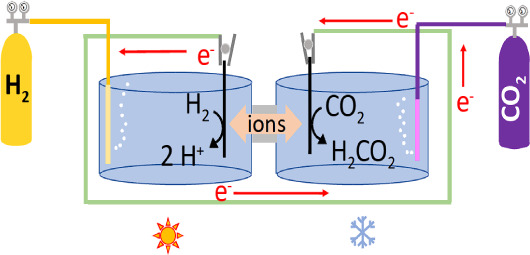

## Introduction

The hydrothermal-vent interface of early submarine vent systems on Hadean Earth (4.5 to 4 billion years before present, Ga) has long been proposed as a hatchery for the emergence of early bioenergetic processes.^1–3^ This model is based on the mechanism of chemiosmosis, or vectorial electrochemistry,^4–6^ which describes a free energy conversion mechanism that is universal in modern cells. In such a cellular mechanism, exergonic and endergonic processes are coupled using natural electrochemical gradients, resulting from pH and redox gradients, as drivers of chemical reactions mediated by membrane-located molecular machines, or enzymes.^7^ This energy conversion system is considered as universal and as ancient as the genetic code in known organisms.^8,9^ In a model of early cell metabolism, a key step for emergence includes the reduction of oceanic CO_2_ by serpentinization-derived H_2_ mediated by vent minerals, in a prebiotic analogy to the reductive acetyl Coenzyme A (acetyl-CoA) pathway (also known as the Wood–Ljunghdahl pathway; WL).^10^ This is considered an important pathway for understanding the early phases of biochemical evolution, as it forms forms the reactive molecule acetyl-CoA, and is linked to the formation of ion gradients used to power cellular processes.^11–14^ From this context, we seek to investigate the first steps for the emergence of a key metabolism for life: the CO_2_ reduction to formic acid and its subsequent reduction to acetic acid, under different conditions proposed for the hydrothermal–vent interface.

This model for the emergence of bioenergetics offers pH and natural electrochemical gradients as driving forces for protobiological processes, facilitated by the interface between hydrothermal fluids and the primitive ocean (which we will refer to as the early vent-ocean interface). In that scenario, vent efflux of moderate temperature (∼70 °C), moderate reducing potential, and alkalinity (pH ∼ 9) juxtaposed with the cold, mildly acidic early oceanic fluids (pH 5.5),^3,15^ both percolate through a labyrinth of micropores in the mineral precipitate that mediates the interface.^16^ The mechanism of catalysis proposed is mediated by iron and sulfur (Fe–S) minerals or by iron oxyhydroxides (green rusts)—both plausible on early Earth in the context of hydrothermal vents.^3,10,17^ Clusters of Fe–S and iron oxides are considered precursors of important enzymes for carbon transduction and fixation processes, such as ferredoxin, used in CO_2_ reduction, and fumarate reductase.^18–20^

The WL pathway depends, like other cellular processes, on iron-sulfur cluster containing proteins which work in concert with the proton gradient. Thioesters, such as acetyl-CoA, are extremely important in prokaryotic metabolisms, such as those of methanogens and acetogens, in transduction pathways.^21–23^ Furthermore, CO_2_ is a solute of relative abundance in the primitive ocean, and H_2_ is a well-known product of serpentinization reactions.^16,24^ Given the universality of the acetyl-CoA pathway, such metabolism may have already existed in the Last Universal Common Ancestor (LUCA).^22,25–27^ The initial and limiting step of this metabolism, the formation of formic acid from CO_2_, was recently demonstrated in the laboratory in the absence of enzymes, using the mediation of metallic samples and under hydrothermal conditions.^26^

This variant of the submarine alkaline vent theory (SAVT) emphasized in this work has already guided experimental investigations that explored the proposed requisite mechanisms of early energy conversion.^2,28–31^ We recently demonstrated CO_2_ reduction to formate by H_2_, which is the first and limiting step of the WL pathway, using microfluidic setups under the conditions of the early vent–ocean interface.^32,33^

These microfluidic studies elucidated important aspects of a possible carbon-fixation mechanism hosted at the early hydrothermal-vent interface. Specifically, they suggest an indirect electrochemical coupling of the CO_2_ reduction process with H_2_ oxidation, which occurs on different sides of the inorganic barrier. Herein, to better probe the mechanism, we employ macroscale reactors in our examination not only for CO_2_ reduction to formate but also for the first C–C bond-forming step in the production of acetic acid. Thus, we aim to simulate different conditions that likely existed in a gradient-rich early alkaline vent scenario, and we investigate the effects of those variables on the limiting steps of the acetyl-CoA pathway using [Ni-]­FeS minerals. The effect of Ni in the mineral structure, the thermal gradient, and the occurrence of different passive and induced electrical currents were tested to better understand how those conditions might have contributed to CO_2_ fixation at early hydrothermal–vent interfaces. Moreover, we also investigated the structure and composition of [Ni-]­FeS minerals formed by coprecipitation, used in a variety of microfluidic protocols.^32–35^ Finally, the macroscale reactor used in this study enables electrochemical monitoring, allowing us to probe the electron-transfer mechanism for the redox reactions involving CO_2_ and H_2_ mediated by the mineral interface.

In order to contextualize our study among prior analogue hydrothermal vent research, we use Figure 1 to compare and contrast various generalized reactor schemes (detailed schematics for ours are available in the Supporting Information). Reduction of CO_2_ under batch hydrogenation conditions (Figure 1A) has been facilitated by metal/mineral particles toward the production of formic acid, methanol, acetic acid, and pyruvate, employing both H_2_ and CO_2_ in one pressurized vessel.^36^ Alternatively, rather than employing H_2_ as a reducing agent, metals and minerals have been used as direct sacrificial reductants, again toward the production of various organics.^27,37,38^ In another alternative to H_2_, a reducing potential condition is controlled by a potentiostat, while metals and minerals are tested as electrocatalysts for CO_2_ reduction (Figure 1B).^19,39,40^ In this scenario, potentiostat-driven electrical current is monitored or set by the potentiostat, and the “H” incorporated into CO_2_ reduction products is derived from the analogue ocean electrolyte. To mimic the driving force for CO_2_ reduction in methanogens and acetogens (harnessing pH gradients and electrochemical potentials across a barrier), we constructed a microfluidic reactor (Figure 1C) in which H_2_ oxidation and CO_2_ reduction appear to have been compartmentalized in different fluids separated by a freshly precipitated mineral barrier.^33^ Electrons from H_2_ oxidation passed the barrier in order to reduce CO_2_ on the other side, but as with the purely electrocatalytic system, the “H” in incorporated products was derived from the analogue ocean fluid rather than the H_2_. We presume that charge balance was maintained by ion passage through the precipitated mineral or by coelution of the ocean/vent fluids. Following up on the microfluidic reactor, we were interested in moving to a macroscale reactor for longer reaction times, for independent control of temperature, and for inclusion of electrodes within the system.

**Figure 1:**
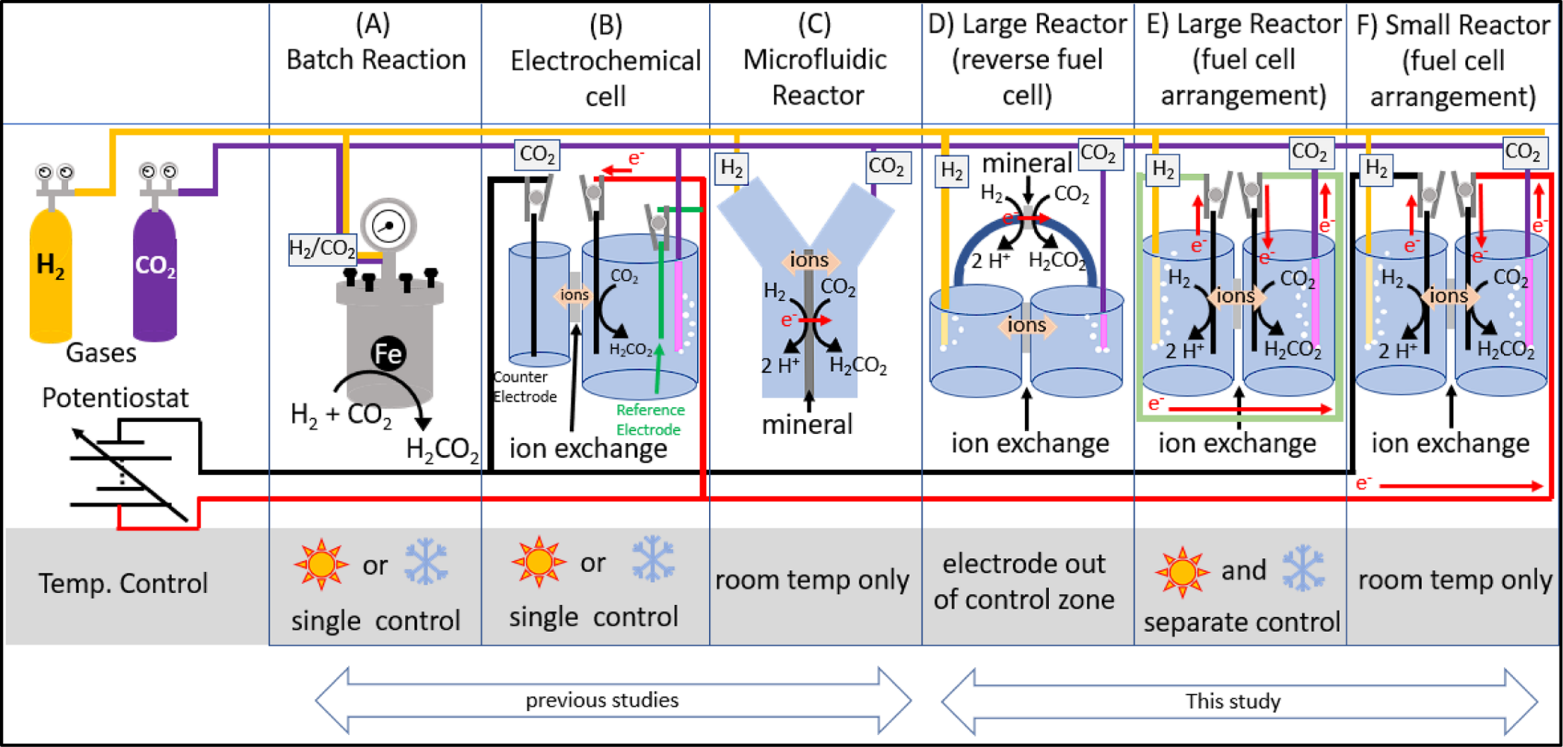
Generalized reactor setups for mineral/metal-facilitated reduction of CO_2_ under analogue hydrothermal vent conditions.

In the present study, we started by developing a ∼500 mL glass reactor, set up with a “reverse fuel cell” arrangement (Figure 1D). The reverse fuel cell arrangement refers to the use of a single solid mineral or mineral-painted metal through which electrons can pass but ions cannot (so we included an ion-conductive membrane to maintain charge balance). This glass reactor included separate water jackets on the “ocean” and “vent” sides for independent temperature control. Although the mineral in the reverse fuel cell arrangement was included between a hose connecting the two reactor halves, it was outside the temperature-controlled zone, so we could not effectively set up a temperature gradient with this arrangement. To achieve a temperature gradient, we opted for a “fuel cell” arrangement, whereby we split the mineral/electrode in two and placed them in both chambers on either side of the ion-conducting membrane. Both setups were sufficient to enable electrons from H_2_ oxidation in the vent side to passively flow to the ocean side to drive CO_2_ reduction. However, given the size of the reactor, the distance that ions needed to travel for charge balance, and the small projected currents from passive/spontaneous reactions, the system overall in either large glass reactor setup was too resistive for our potentiostat to measure the currents generated. In order to monitor or control currents, we constructed a smaller (∼20 mL) PTFE reactor, again in the fuel cell arrangement separated by an ion-conductive membrane (Figure 1F). This system did not allow for independent temperature control, but the smaller volume and shorter path between electrodes produced a less-resistive system in which our potentiostat could either control or measure currents between the two chambers.

## Methods

### Materials and Solutions Preparation

All solutions were prepared with high-purity water (Milli-Q system, 18.2 MΩ·cm) and with reagent-grade chemicals. The main electrolytes used, the vent analog and ocean analog fluids, were based on works from the literature (Table S1).^10,32,33^ For saturation of solutions, CO_2_ and H_2_ gases (purity: 99.99%) were used. Additional information on methods can be found in the Supplementary Information (SI).

### Synthetic Minerals Preparation

For the synthesis of FeS and Ni-FeS, a coprecipitation method, also based on works from the literature, was used. In this work, mineral-formation fluids (Table S2) were mixed in a 1:1 proportion to form precipitates of Fe–S and Ni–Fe–S minerals. Then, the resulting suspension was separated by centrifugation, and the precipitated mineral paste was used as an electron-conductive ink, which was painted onto Fe (>98%) electrodes.

### Synthetic Minerals Analyses

The synthesized minerals were characterized via powder X-ray diffraction (p-XRD), X-ray fluorescence (XRF), Raman microscopy, scanning electron microscopy (SEM), energy-dispersive X-ray spectroscopy (EDX), and the Brunauer–Emmett–Teller (BET) method.

### Experimental Setups

For experiments investigating pH- and redox-gradient-driven CO_2_ reduction mediated by different minerals and the effect of thermal gradients, we used a two-chamber glass reactor with separated water jackets and a capacity of 250 mL in each chamber (Figure S1). To simulate the vent-ocean interface, a vent analogue fluid and an early ocean analogue were used as electrolytes (Table S1). To check the gradient-driven formation of organics, natural pyrite (FeS_2_) pieces or Fe pieces painted with a mineral ink mediated the interface in contact with both fluids simultaneously in a reverse fuel cell arrangement (Figure S1a). An ion-exchange membrane (Nafion 212), pretreated in 0.1 M NaOH, separated the two chambers for ionic equilibration during the experiments. The fluids within the reactors were degassed for at least 1 h by sparging with CO_2_ (ocean side) and H_2_ (vent side) prior to each experiment, and bubbling persisted at atmospheric pressure throughout the duration of each experiment.

For analyzing thermal-gradient effects, the large glass reactor was used in the fuel cell arrangement (Figures 1E and S1a), with separate temperature control for the two compartments (vent/ocean: 75 °C/75 °C; 75 °C/6 °C 6 °C/6 °C). In this setup (Figure S1b), Fe pieces painted with a mineral were immersed in each side of the reactor, at a proper distance to maintain a temperature gradient between each electrode. The same electrolytes, ion-exchange membrane between each reactor chamber, and gas saturation method were used as in the reverse-fuel-cell arrangement.

To directly electrochemically monitor the system with a potentiostat, we used a smaller reactor with a shorter distance between electrodes and, thus, less overall resistivity. This two-chamber PTFE reactor had a 20 mL capacity and a magnetic stirrer in the ocean side (Figure S2). The same electrolytes, ion-exchange membrane, and minerals used with the large glass reactor were also used here. CO_2_ and H_2_ gases were thoroughly bubbled in the ocean side and vent side, respectively, for at least 30 min prior to each experiment, and balloons were used to maintain a saturated headspace during each experiment. For these experiments, we induced between the two electrodes fixed voltages (−0.5 and −2 V) as well as fixed currents of −1 nA and −10 μA (negative indicates that the electrons flow toward the working electrode on the ocean side).

For all of the experiments described, the organic aqueous product analyses were performed via proton nuclear magnetic resonance (^1^H NMR).

## Results

### Organics Formation Mediated by Different [Ni-]­FeS Minerals

Screening of various minerals indicated that FeS, Ni-FeS, and FeS_2_ all produced short-chain carboxylic acids in μM concentrations on the ocean side of the glass reactor in the reverse fuel cell arrangement at 25 °C (Table 1, entries 1–3), while none was detected on the vent side (Table 1, entry 4). This suggests that, rather than gases or organic molecules passing between chambers, there is an indirect electrochemical mechanism whereby H_2_ is oxidized on the “vent” side of the reactor, electrons pass through the mineral, and this current reduces organics on the “ocean” side of the reactor. Control experiments with no pH gradient produced neither formic acid nor acetic acid. This suggests that under ambient temperature (∼22–25 °C) and anoxic conditions, the mineral cannot be significantly sacrificially oxidized as the source of electrons for CO_2_ fixation, nor can it effect such a reaction within a system with CO_2_ and H_2_ in the absence of a sufficient pH/redox gradient.

**Table 1:** Organics Formed from Different Minerals Mediating the Early Vent–Ocean Interface Under Different Conditions After 24 H

		Ocean side	Vent side		
Entry	Mineral	gas, pH, T/°C	gas, pH, T/°C	Formic acid (μM)	Acetic acid (μM)
1	FeS_2_ ^a^	CO_2_, 5.7, 25	H_2_, 11, 25	4.2	2.5
2	Ni-FeS^a^	CO_2_, 5.7, 25	H_2_, 11, 25	3.9	n.d.
3	FeS^a^	CO_2_, 5.7, 25	H_2_, 11, 25	n.d.	13
4	FeS^b^	CO_2_, 5.7, 25	H_2_, 11, 25	n.d.	n.d.
5	Ni-FeS^a^	CO_2_, 6.5, 25	H_2_, 6.5, 25	n.d.	n.d.

a Reaction conditions: Large glass reactor. Ocean side fluid: Na_2_Si_3_O_7_ 10 mM in deionized water (purged with CO_2_). Vent side fluid: Na_2_S 100 mM, K_2_HPO_4_ 10 mM, Na_2_Si_3_O_7_ 10 mM (purged with H_2_). Reactor set up with a nafion membrane separating the two cells of the reactor, and single piece of mineral or mineral plated electrode placed between a glass joint, which is connected to each sample compartment by a fluoroelastomer tube. Sample taken from “ocean side” of reactor.

b Sample taken from vent side rather than ocean side. Otherwise, same as conditions provided in footnote a. **n.d.:** None detected.

### Effects of the Thermal Gradient

Using the glass reactor in a fuel cell arrangement (Figure S1b), we screened the effect of temperature on both chambers. With both chambers at 75°C (Table 2, entry 1), formic and acetic acids were observed in much higher quantities compared to results at room temperature (Table 1, entry 2). When both chambers were cooled to 6 °C, none were detected (Table 2, entry 2). Under a temperature-gradient scenario similar to Hadean vent models, the yields for formic and acetic acids (Table 2, entry 3) nearly matched those of all-hot tests. Coupled with the all-hot (slightly higher organic formation) and all-cold (no products detected) tests, the temperature gradient results suggest that higher temperatures on the vent side increase the rate of H_2_ oxidation (the reaction cannot proceed with a “cold” vent side). The ocean side can be either “hot” or ″cold” and still produce significant short-chain carboxylic acids. Perhaps the thermodynamic effect of temperature reduction on the ocean side is countered by a kinetic advantage of increased concentrations of CO_2_ at colder temperatures.

**Table 2:** Organics Formed After 18 h in Simulated Early Vent–Ocean Interface Under Different Temperature conditions^a^

Entry	Mineral	Ocean side gas, pH, T/°C	Vent side gas, pH, T/°C	Formic acid (μM)	Acetic acid (μM)
1	Ni-FeS	CO_2_, 5.7, 75	H_2_, 11, 75	815.8	33.54
2	Ni-FeS	CO_2_, 5.7, 6	H_2_, 11, 6	n.d.	n.d.
3	Ni-FeS	CO_2_, 5.7, 6	H_2_, 11, 75	735.9	11.15

a Reaction conditions: Large glass reactor. ocean side fluid: Na_2_Si_3_O_7_ 10 mM in deionized water (purged with CO_2_). Vent side fluid: Na_2_S 100 mM, K_2_HPO_4_ 10 mM, Na_2_Si_3_O_7_ 10 mM (purged with H_2_). Reactor set up with a nafion membrane separating the two cells of the reactor, and a mineral-painted Fe electrode on both sides, which were connected by a wire. Sample taken from “ocean side” of reactor. **n.d.:** None detected.

### Synthetic Minerals Analyses

Our process of bulk precipitation for mineral formation is analogous to the processes in hydrothermal vent systems, producing porous inorganic barriers that form vent mounds.^1,41,42^ The p-XRD analyses of these minerals show a typical profile for amorphous or nanocrystalline structures that are commonly formed as precursors to FeS minerals like mackinawite^43^ (see Supporting Information). This lack of crystallinity may be due to the simultaneous formation of several phases. Since the focus here was to simulate the natural precipitation of minerals in early hydrothermal systems, we did not seek to control the kinetic or thermodynamic parameters or subtle solution mixing, which play a key role in controlling the growth and shape of crystals.^44^ SEM-EDX (Figures S4 and S5) and XRF (Figure S6) all suggest similar stoichiometric ratios of metal (Fe or Fe/Ni) to S present in the precipitated minerals. Each of these analyses conducted on Ni-FeS indicates a 1–4% doping of Ni within the FeS structure. SEM observations of FeS and Ni-FeS particles show a size range of 1–40 μm (Figure 2). BET analysis indicated a surface area of 6.6 m^2^/g. Given the small doping percentage of Ni within the FeS, the Raman spectra for these two samples are similar, and both are consistent with mackinawite (Figure 3a,b). The Raman spectra for FeS_2_ are consistent with reference materials (Figure 3c).

**Figure 2:**
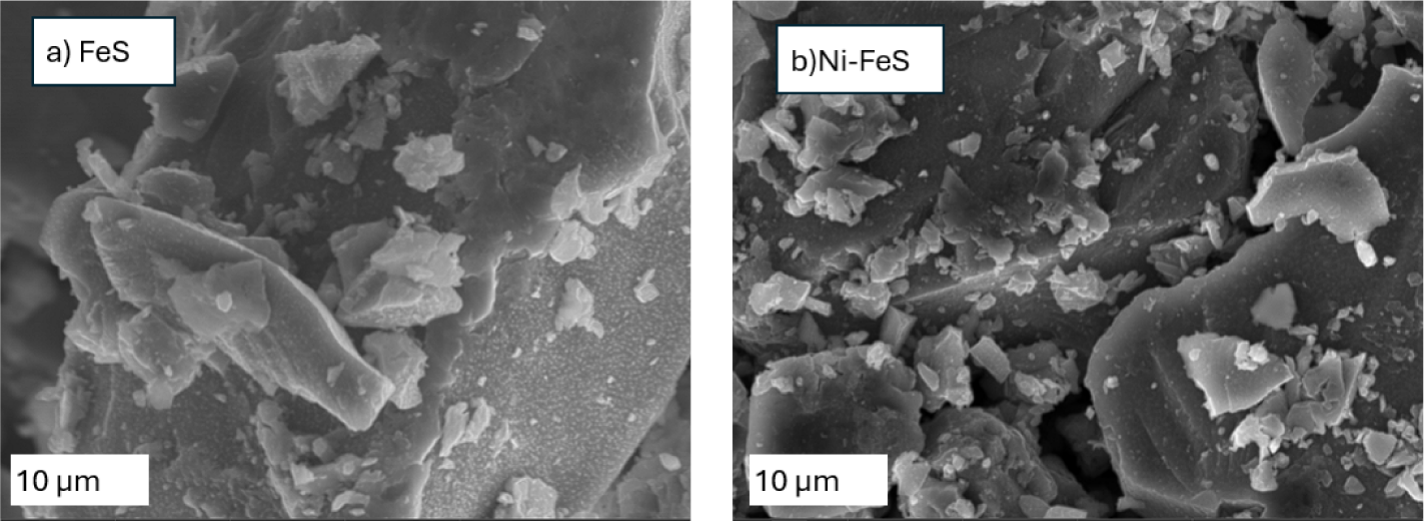
SEM secondary-electron images of (a) FeS and (b) Ni-FeS synthetic minerals, produced using the same procedures as those used in our experiments.

**Figure 3:**
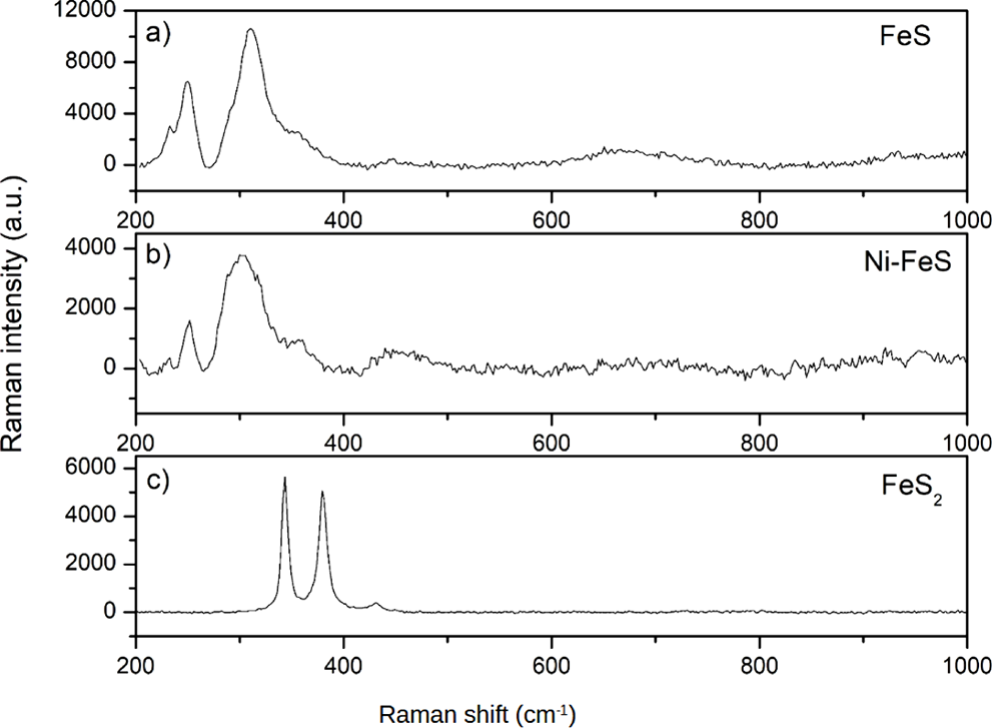
Raman spectra at 532 nm of (a) FeS, (b) Ni-FeS, and (c) FeS_2_.

### Current Control and Second Protometabolic Step

To simulate and investigate specific electrochemical conditions at the vent–ocean interface, experiments were performed within the range of electrochemical conditions expected to exist in natural scenarios. A series of externally induced experiments were conducted under two constant-current conditions: −10 nA (nanoamp) and −10 μA (microamp). Currents of nanoamp magnitude are estimated to exist in our system under spontaneous conditions, which means that the pH and redox gradients naturally induce an electrochemical potential. This estimation was based on the measurement of the current while the system was under open-circuit voltage. We also tested two conditions under an induced fixed voltage: −500 mV and −2.0 V (Table 4). Currents in the range of hundreds of millivolts are also detected in natural hydrothermal systems and are estimated to have existed at the interface of early hydrothermal scenarios.^45,46^ All of the reference conditions were set, considering the mineral on the ocean side as the working electrode.

Ni-FeS showed higher organic product formation and overall faradaic efficiency for CO_2_ reduction relative to FeS under the same electrochemical conditions (Table 3). This indicates that Ni atoms enhance the interaction of FeS with CO_2_ for its electrochemically induced reduction, as shown in other works.^6,47,48^ Analogously, for acetic acid formation, Ni-FeS mediation also shows higher performance in mediating its formation. However, a different pattern appears under the condition of a fixed voltage of −500 mV (Table 4, entries 1 and 3), where FeS shows a greater capacity to form formic and acetic acid. This altered pattern was detected in formic acid production at −2.0 V. In general, by several measures, pristine FeS should outperform Ni-FeS. DFT calculations suggest that Ni doping of FeS limits its catalytic activity toward CO_2_ reduction.^49^ Additionally, our synthesized Ni-FeS is less electrically and ionically conductive compared to our synthesized FeS minerals.^50^ Since our Raman analyses and SEM observations suggest similar structures and show similar particle sizes/morphologies, these variables seem unlikely contributors to the enhanced catalytic activity of Ni-FeS over FeS. Ni doping has been shown to increase the stability of FeS,^51,52^ so the relative stability of the Ni-FeS mineral may have more to do with its superior performance than the lower activation energies and higher conductivities associated with FeS.

**Table 3:** Results of Organic Formation After 12 h from Current-Induced Experiments Using PTFE Reactor Setup (Figure S2) Under Room Temperature^a^ ^,^ ^b^

			Ocean side	Vent side	Formic acid		Acetic acid	
Entry	Mineral	Current induced	gas, pH	gas, pH	Concentration (μM)	Faradaic efficiency	Concentration (μM)	Faradaic efficiency
1	FeS	10 nA	CO_2_, 5.7	H_2_, 11	5.56	20.67%	6.40	8.8%
2	FeS	10 μA	CO_2_, 5.7	H_2_, 11	n.d.	0%	4.86	0.005%
3	Ni-FeS	10 nA	CO_2_, 5.7	H_2_, 11	8.84	32.77%	43.23	29.05%
4	Ni-FeS	10 μA	CO_2_, 5.7	H_2_, 11	12.70	0.05%	12.41	0.012%
5	FeS^b^	-	CO_2_, 5.7	H_2_, 11	1.26	-	0.43	-
6	Ni-FeS^b^	-	CO_2_, 5.7	H_2_, 11	34.37	-	11.22	-
7	Fe^b^	-	CO_2_, 5.7	H_2_, 11	n.d.	-	n.d.	-

a Reaction conditions: 10 mL PTFE reactor (Figure S2). Ocean side fluid: Na_2_Si_3_O_7_ 10 mM in deionized water (purged with CO_2_). Vent side fluid: Na_2_S 100 mM, K_2_HPO_4_ 10 mM, Na_2_Si_3_O_7_ 10 mM (purged with H_2_). Reactor set up with a nafion membrane separating the two cells of the reactor, and a mineral-painted Fe electrode on both sides, which were mediated by a potentiostat. Sample taken from “ocean side” of reactor.

b Spontaneous system. Minerals or metals were connected by an electron-conductive wire. **n.d.:** None detected.

**Table 4:** Results of Organic Formation After 12 h from Voltage-Induced Experiments Using PTFE Reactor Setup (Figure S2) Under Room temperature^a^

			Ocean side	Vent side	Formic acid		Acetic acid	
Entry	Mineral	Voltage induced (V)	gas, pH, T/°C	gas, pH, T/°C	Concentration (μM)	Faradaic efficiency	Concentration (μM)	Faradaic efficiency
1	FeS	–0.5	CO_2_, 5.7, 25	H_2_, 11, 25	667.54	0.74%	17.32	0.005%
2	FeS	–2.0	CO_2_, 5.7, 25	H_2_, 11, 25	834.02	0.20%	30.77	0.0019%
3	Ni-FeS	–0.5	CO_2_, 5.7, 25	H_2_, 11, 25	762.31	0.29%	19.50	0.0019%
4	Ni-FeS	–2.0	CO_2_, 5.7, 25	H_2_, 11, 25	558.49	0.10%	30.96	0.0022%

a Reaction conditions: 10 mL PTFE reactor (Figure S2). Ocean side fluid: Na_2_Si_3_O_7_ 10 mM in deionized water (purged with CO_2_). Vent side fluid: Na_2_S 100 mM, K_2_HPO_4_ 10 mM, Na_2_Si_3_O_7_ 10 mM (purged with H_2_). Reactor set up with a nafion membrane separating the two cells of the reactor, and a mineral-painted Fe electrode on both sides, which were mediated by a potentiostat. Sample taken from “ocean side” of reactor. **n.d.:** None detected.

Additionally, under a nanoamp current, the faradaic efficiencies were much higher for organic production, mainly for Ni-FeS. Although we did not mechanistically probe how Ni enhanced reactivity, prior work has demonstrated how specific currents, not necessarily the higher ones, may result in better efficiencies when they result from different potentials; they often show a non-linear relation with reaction yields, as commonly seen in electrochemistry works showing faradaic efficiencies over different potentials.^6,53,54^ Beyond simple CO_2_ reduction, two different single-carbon precursors must couple for the formation of the two-carbon acetic acid. Some currents may favor the formation of CO_2_ reduction products that are not reactive C1 coupling partners for acetic acid formation. For example, the drop in acetic acid formation for Ni-FeS from a 10 nA current (43 μM; Table 3, entry 3) to a 10 μA current (12 μM; Table 3, entry 4) could be the result of the shunting of otherwise reactive CO_2_ reduction products into something like methane or ethane (gas-phase products that we are not set up to observe and that is not a likely coupling partner toward the production of acetic acid).^54^

Considering the well-known sensitivity of iron sulfides to changes in local electrochemical conditions,^55–58^ we expect that different phases may exist under the experimental conditions tested here, as occurs under analogous conditions.^59^ Key enzymes in the WL pathway are also known to be sensitive to O_2_ and electrochemical conditions.^17^ A specific mineral phase or cluster may have facilitated the formation of acetic acid. Moreover, the experiments conducted here were performed in a reactor designed to simulate the vent–ocean interfacial electrochemical conditions, and the long period of residence for the experiments may also be the reason for the detection of acetic acid, which is not detected in other setups.^10,33^ Moreover, different phases show greater stability under different potentials, and some of these may prevail after the 12 h of experiments, which can explain the distinct patterns observed under −500 mV and −2 V.

Notably, small amounts of organics were detected in the experiment performed under thermodynamically spontaneous conditions with no external electrochemical induction. In this experiment, two mineral-coated iron pieces are directly connected by an electron-conductive wire, without the mediation of the potentiostat (Figure S2b), thus, only pH and redox gradients are acting. The fact that the formic acid and acetic acid yields in these closed-circuit results (Table 3, entries 5 and 6) are closest to those with an applied nanoamp current suggests that, in this passive system, nanoamp-scale current may be all that is passing through the mineral mediators. Nevertheless, these systems efficiently enable higher yields of CO_2_ reduction.

The lack of reactivity for our polished, bare iron electrode (Table 3, entry 7) is consistent with other purely electrochemical investigations, which suggest that iron metal is an ineffective electrocatalyst for CO_2_ reduction.^60,61^ However, other studies have demonstrated iron as an effective sacrificial reducing agent for CO_2_ reduction, so a comparison of surface area is important.^27,37^ Under pressurized (MPa) batch conditions at elevated temperatures (at least 80 °C), He et al.^37^ demonstrated the CO_2_ reduction by oxidation of iron nanoparticles. Given the amount of iron (5 mmol) and the size of the particles (200 nm), we calculate the surface area in their system available for sacrificial oxidation to be 1060 m^2^/g. With their mildest tested conditions (which would still represent an overestimation based on our much milder ambient temperature/pressure conditions), He and coauthors measured 1 mmol of formic acid produced under a pressure of 1.4 MPa CO_2_ and 80 °C after 72 h. This equates to a formate production of 14 nm/m^2^ hr. Considering the 5,000,000-fold smaller geometric surface area of our bare iron electrodes (2 × 10^–4^ m^2^), it is understandable that we do not observe formate production even by this sacrificial oxidation route. Given our available geometric surface area, this would translate to 67 picomoles of formate produced in the 24 h reaction period, which would likely be below our detection limit.

While sacrificial oxidation is a plausible route to CO_2_ reduction with bare iron (albeit not substantial enough to produce detectable levels of formate under our conditions), electrocatalysis is the only viable route for formate production in other tests. For example, FeS_2_ is a thermo-oxidatively robust mineral that is stable under a wide Eh/pH range, so any production of formate mediated by this mineral must be the result of electroreduction at the surface of the pyrite electrode. Because this electrode is a single, solid, polished, smooth piece of pyrite, we consider only the geometric surface area of the exposed mineral (2.67 × 10^–4^ m^2^). Therefore, the lower rates of organic production for FeS_2_ compared to FeS/Fe­(Ni)S may be the result of a higher fundamental catalytic activity for FeS/Fe­(Ni)­S, or may simply be the result of significantly higher surface areas for the latter (applied as a paint of micrometer-sized particles). Given the BET surface area analysis for Fe­(Ni)S (6.6 m^2^/g), and the tens of miligrams applied through electrode painting, we estimate the available specific surface area of these minerals to be up to 200-fold higher than the polished smooth geometric surface area of the FeS_2_.

## Discussion

### Effect of Thermal Gradient for the Energy Protometabolism at Early Hydrothermal Systems

Thermal gradients are known to exist in modern cellular systems, but it is still under debate whether they are crucial or what their exact function is for life. A 10°C gradient has been detected across mitochondrial membranes.^61,62^ Under interface conditions, the thermal gradient results in an electrochemical potential, the well-known Seebeck effect/voltage. The potential generated from that effect is usually considered irrelevant, as it generally ranges from μV to hundreds of millivolts per Kelvin. On the other hand, thermodynamic and kinetic parameters of chemical reactions hosted in an interface region may also be considerably affected by temperature gradients. The vent-ocean interface modeled in the SAVT and evidenced by experimental work hosts reactions that are coupled on both sides of the interface, and these reactions can be independently affected by the local temperature on each side of the interface. The two reactions are represented below:

$$kCO_{2}\left(\mathrm{aq}\right)+n\left(H^{+}+e^{-}\right)⇄P+mH_{2}O\left(l\right)$$

$$H_{2}\left(\mathrm{aq}\right)+2OH^{-}⇄2H_{2}O\left(l\right)+2e^{-}$$

$${\{P=\mathrm{HCOOH},\mathrm{CH}}_{3}\mathrm{COOH},\mathrm{HCHO}\}$$

In the early vent–ocean interface, both reactions are coupled and mediated by an inorganic electron-conductive [Ni-]­FeS mineral. Figure 4 shows the results of an analytical model inspired by the works of Herschy et al.,^10^ and Ooka et al.,^30^ adapted to the context of the early vent-ocean interface. Based on those results , it is clear that thermodynamics become more favorable when there is a higher temperature gradient, as it increases the potential of both coupled reactions.

**Figure 4:**
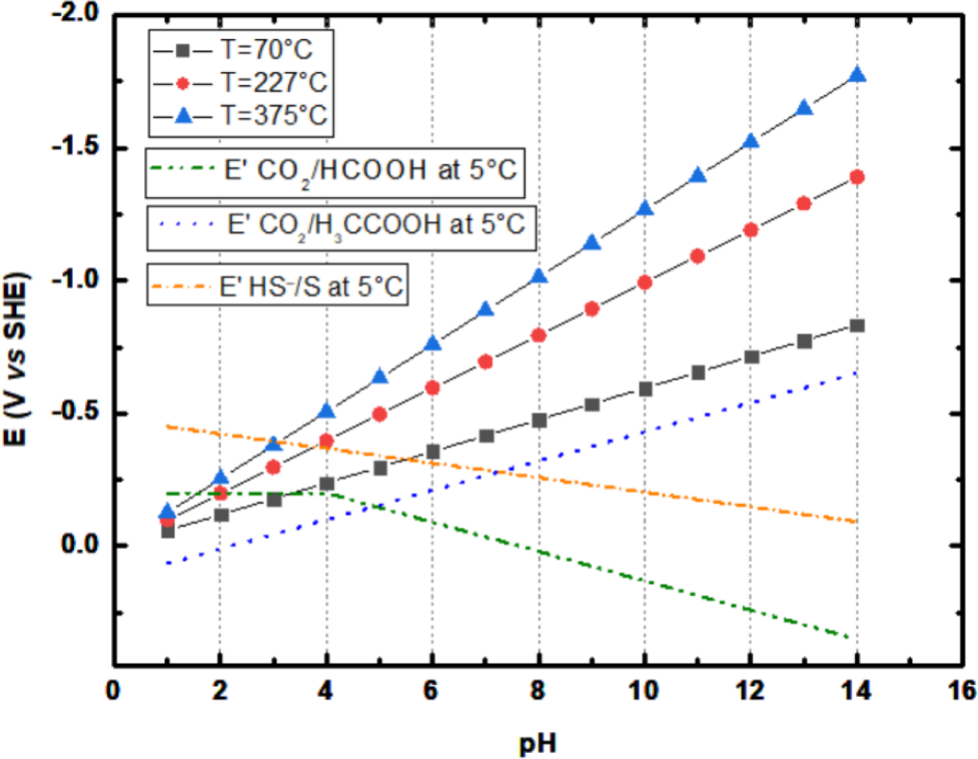
Equilibrium potentials for the H_2_ oxidation reaction (triangles, circles, and squares) at different temperatures based on different geological environments. The reference of the potentials calculated is the standard hydrogen electrode (SHE) Considering *T* = 70 °C detected in fluids of Lost City Hydrothermal Fields (LCHF)^63^ and the natural radioactive environment in Witwatersrand basin,^64^ *T* = 227 °C for the Okinawa Trough hydrothermal field,^30^ and *T* = 375 °C for the Rainbow hydrothermal vent.^24^ It is also shown the HS^–^/S redox pair potential (dashed yellow line) over pH, and the potential required for reduction of CO_2_ to HCOOH (dashed green line), based on the work of Kortlever et al.,^65^ and to HCCOOH (dashed blue line) at the temperature of 5 °C.

The model that results in Figure 4 illustrates the overall thermodynamic advantage that the thermal gradient may promote. On the other hand, there is evidence of the vent–ocean interface kinetics from the results of the all-cool and all-hot experiments. While, under low temperatures, H_2_ and CO_2_ gases are expected to be more soluble, and the high concentration can increase the thermodynamic parameters; however, it can also decrease the reaction rates. Nevertheless, the reaction rates are expected to increase at higher temperatures, although the gas concentrations decrease. Thus, the organic formation detected under the all-hot conditions is higher in concentration even compared to the experiments under thermal-gradient conditions. Additionally, the fact that there were no organics under “all-cool” conditions leads us to the conclusion that kinetic control should prevail in the modeled system.

### Outcomes for the Emergence of Life Model in Early AHV Systems

Modern hydrothermal systems show natural electrical currents and local reducing conditions on surfaces that also show different potential and current in different regions.^45,46,66^ Models of early alkaline hydrothermal systems also showed how electrochemical parameters might be sensitive to the local composition of the early ocean and, consequently, to the mineral barrier composition.^67^ Here, we demonstrate how the Ni-FeS mineral formation condition in alkaline hydrothermal systems results in a mixture of phases that cannot be easily identified. Under different electrochemical potentials, the prevailing [Ni-]­FeS mineral phases can also change, as shown in recent experimental works, which may promote the formation of a metallic phase.^11,39,68^ On the other hand, the low oxygen fugacity (fO_2_) and controlled sulfur fugacity (fS) in our systems might have contributed to the local stability of the mineral sulfide phases during our experiments. That is analogous to conditions in early hydrothermal vent systems, which were expected to have had low fO_2_ due to serpentinization that reduces water, forming hydrogen gas.^69,70^

The gradient-rich environment, along with the diversity of mineral phases at the vent–ocean interface, may be a key factor that makes the AHV scenario into a hatchery for life on early Earth, as envisioned in several works.^71–75^ As concluded from the discovery of the lamellar distribution of minerals in black smokers, the inner layer is dominated by chalcopyrite in contact with hydrothermal fluid, while the outside layer is dominated by pyrite (FeS_2_) and sphalerite (Zn, Fe)S in contact with seawater. Inside that structure, the mineral layers are composed of pyrrhotite (FeS), pyrite, and sphalerite. A gradient structure such as this is expected to affect the catalytic properties at submarine vent interfaces.^76^

As shown here, after a period of hours, both formic acid and acetic acid are present in detectable concentrations under vent-ocean interface conditions. This indicates the possibility that at least two protometabolic steps could occur in the early vent-ocean interface, as illustrated in Figure 5. The variation of electrochemical conditions expected to exist in early hydrothermal systems, summed with the variation of Ni-FeS mineral phases, does not compromise the occurrence of those protometabolic steps but seems to only affect the accumulation of formic acid, probably due to the kinetics of the first step.

**Figure 5:**
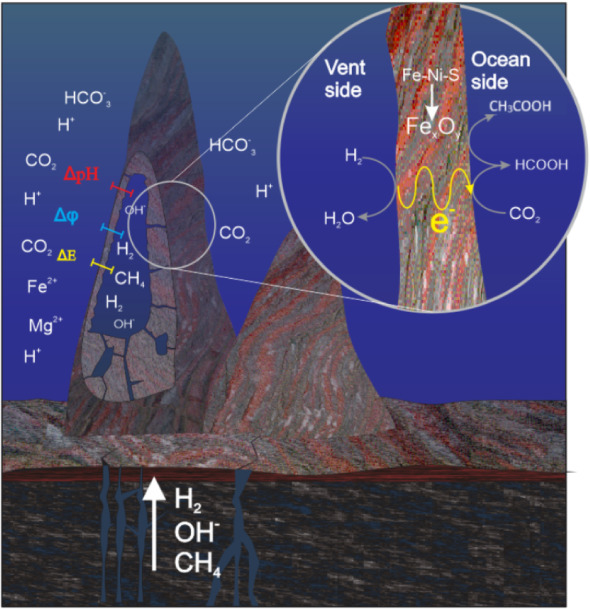
Sketch of the free-energy transfer mechanism from natural gradients at the early vent–ocean interface to drive the energy protometabolism, a candidate seed to the chemiosmosis’ emergence on early Earth. Represented in the figure are the different gradients in the vent–ocean interface: the pH (ΔpH) , redox (Δφ) , and the resulting potential gradient (ΔE) . The Ni-FeS-rich barrier mediates the electron-transfer mechanism coupling the H_2_ oxidation and the CO_2_ reduction in a WL-like protometabolism where two steps occur in the vent–ocean interface.

Moreover, we can expect that another factor can change the production of formic acid at the interface: the thermal insulation of the inorganic barrier. As shown here, a warmer temperature on both sides of the vent–ocean interface contributes to higher rates of CO_2_ electrochemical reduction. This condition is expected to occur when thermal insulation is low enough, which can occur in regions of the vent system with thin barriers or closer to regions where serpentinization is occurring. On the other hand, in distant regions or where there is low serpentinization activity, thus cooler temperatures, very little CO_2_ reduction can be expected, despite the presence of natural electrochemical gradients. In cases of high thermal insulation of the mineral, a temperature gradient (hot vent/cool ocean) is nearly as effective as the all-hot scenario.

While higher overall product formation can be seen at higher currents (10 microamperes), excellent faradaic efficiencies can be achieved with an approximation of the passive/expected current (10 nanoamperes). Herein, we demonstrate that a very small current can be extremely efficient for CO_2_ reduction.

## Conclusions

This investigation into mineral-facilitated electroreduction of CO_2_ suggests conditions under which reactions from a protometabolic Wood-Ljungdahl pathway may be viable. Formic acid and acetic acid can be generated in μM quantities by the passive flow of electrons following geologically plausible pH and redox gradients established at hydrothermal vents. Higher temperatures lead to better conversion of CO_2_ to formic acid, although the reaction can still proceed under a temperature gradient analogous to that of extant biological systems and similar to those produced in ocean vents. Extremely small currents (nanoamps) with pH and redox gradients can efficiently enable CO_2_ reduction. These results support the SAVT model while narrowing the search for optimal conditions for important protometabolic steps in the potential emergence of life. Our results also suggest that a variety of mineral mediators, in the presence of a gradient-rich vent environment, can induce two electrochemically mediated metabolic steps for energy conversion and carbon fixation in the absence of enzymes at the interface of alkaline hydrothermal systems.

## Supplementary Material







## Notes

SEM, Raman spectroscopy, XRF, p-XRD, and NMR analyses are included. Detailed experimental procedures and reagents along with reactor schematics are included as well. (PDF)
Raw data collected from NMR analyses (ZIP)
Raw data collected from experiments conducted using a potentiostat (ZIP)

## Abbreviations

WL: Wood–Ljungdahl
NMR: Nuclear Magnetic Resonance
AHV: Alkaline Hydrothermal Vents
SAVT: Submarine Alkaline Vent Theory
XRD: X-ray diffraction
XRF: X-ray fluorescence
SEM: Scanning Electron Microscope
EDX: Energy dispersive
