# Supplementary material for: Carbon Reduction Powered by Natural Electrochemical Gradients under Submarine Hydrothermal Vent Conditions
Source: J Am Chem Soc. 2025 Jul 29;147(32):28674–83. doi: 10.1021/jacs.5c01948 (PMC12356197; doi:10.1021/jacs.5c01948)
Supplement: Supplementary file 1 [file ja5c01948_si_001.pdf]

Supplementary Information

Carbon reduction powered by natural electrochemical gradients under submarine hydrothermal vent conditions

T. Altair[1,2*], E. Dragoti[1], V. Sojo[3], Y. Li[4,5], S. Skiffington[6], W.A. Sullivan[7], G.T. Drozd[6], S.E. McGlynn[4,5,8], D. Galante[9], H. Varela[2*], R. Hudson[1,6*]

AUTHOR ADDRESS. [1]College of the Atlantic, Bar Harbor, Maine 04609 USA, [2] São Carlos Institute of Chemistry, University of São Paulo, São Carlos 13560-970, Brazil, [3]Institute for Comparative Genomics & Richard Gilder Graduate School, American Museum of Natural History, New York, New York 10024, USA, [4] Earth-Life Science Institute, Institute of Science Tokyo, Tokyo 152-0033, Japan, [5] Center for Sustainable Resource Science, RIKEN, Wako 351-0198, Japan, [6]Department of Chemistry, Colby College, Waterville, Maine 04901, United States, [7]Department of Geology, Colby College, Waterville, Maine 04901, USA, [8] Blue Marble Space Institute of Science, Seattle, Washington 98104, USA, [9]Institute of Geosciences, University of Sao Paulo, Sao Paulo 05508-080 Brazil.

MATERIALS AND EQUIPMENTS USED

Materials and solutions preparation. All solutions were prepared with high purity water (Milli-Q system, 18.2 MΩ.cm) and with reagent grade chemicals. The main electrolytes used in all experiments, the vent analog and ocean analog fluids, was based on works form literature [1–3]. In Table S1, there is the concentration of both fluids, the ocean-analog fluid composed of $Na_2Si_3O_7.9H_2O$ (supplier) and the vent-analog fluid, composed of Na_2S (supplier), K_2HPO_4 (supplier), and $Na_2Si_3O_7.9H_2O$ (supplier). For saturation of solutions, CO_2 and H_2 gases (Maine Oxy, highly pressurized, purity: 99.99%) were used.

Table S1. Chemicals and concentrations used for the electrolytes that simulate the early vent-ocean interface

Electrolyte	Ocean-analog fluid	Vent-analog fluid
Composition	$Na_2Si_3O_7$ 10 mM	Na_2S 100 mM<br>K_2HPO_4 10 mM<br>$Na_2Si_3O_7$ 10 mM
Gas purged	CO_2	H_2
Temperature	~5.5°C	~65°C

Synthetic minerals preparation.

Table S2. Chemicals and concentrations used for the [Ni-]FeS mineral syntheses via co-precipitation

Mineral synthesized	FeS		Ni-FeS	
**Solution**	**A**	**B**	**A**	**B**
Composition	$FeCl_2$ 50 mM	Na_2S 100 mM<br>K_2HPO_4 10 mM<br>$Na_2Si_3O_7$ 10 mM	$FeCl_2$ 50 mM<br>$NiCl_2$ 5 mM	Na_2S 100 mM<br>K_2HPO_4 10 mM<br>$Na_2Si_3O_7$ 10 mM

Electrochemistry setup. The potentiostat-induced experiments were performed with a two-electrode setup connected to a mini-potentiostat (Admiral Squidstat Plus). The mineral in the "ocean side" was the working electrode while the one in the "vent side" was the counter/reference electrode. The electrochemical cell reactor in this set of experiments was a 20-ml PTFE reactor with two chambers with a magnetic stirrer in the ocean side (Figure S2a). Same electrolytes, ion exchange membrane and minerals used with the large glass reactor (Figure S1) were used here.

For all the experiments described, the organic aqueous product analyses were performed via proton nuclear magnetic resonance (1H-NMR) and ^{13}C NMR (Bruker 500 MHz Ascend NMR Spectrometer).

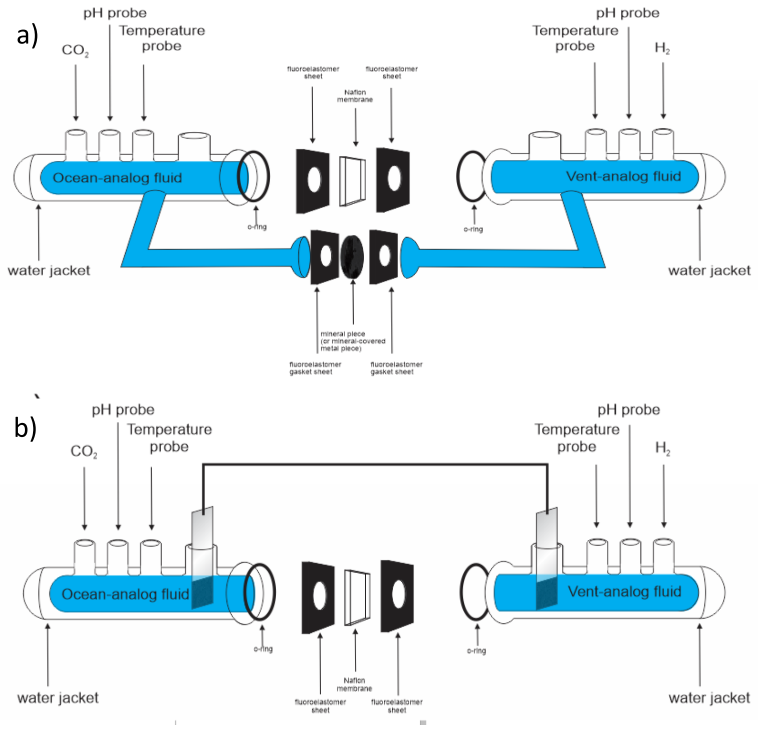

Figure S1. Glass electrochemical reactor setup used for CO_2 reduction driven by natural gradient generated in a simulated early vent-ocean interface. Two physical arrangements were tested: (a)A reverse fuel cell arrangement, where one using two pieces of Fe-S-electroplated iron in each compartment; and (b) A fuel cell arrangement, where using a single mineral-coated metal piece.

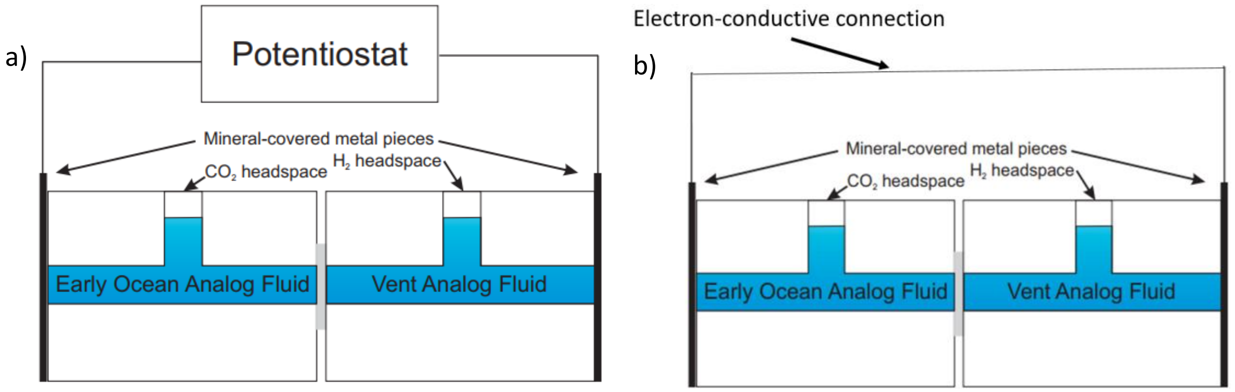

Figure S2. Homemade PTFE reactor with stirring system used for (a) potentiostat-induced experiments under room temperatures (b) spontaneous gradient-driven control experiments.

Mineral analyzes. Analyzes were performed via powder X-ray diffraction (p-XRD, Bruker D2 Phaser Benchtop X-ray Powder Diffraction), Raman spectroscopy (Renishaw inVia), scanning electron microscope coupled with Energy-dispersive x-ray spectroscopy (SEM-EDX), and X-ray diffraction (XRF, Bruker M4 Tornado micro-XRF).

Raman spectroscopy. Raman spectroscopy was performed using a Renishaw inVia Qontor Confocal microscope. The Raman scattering light source was a diode-pumped solid-state laser, 50 mW at 532 nm, operated at 1-10% power. Spectra were collected using a 20X ultra-long working distance objective (Olympus) followed by an 1800 line/mm dispersive grating and a Peltier-cooled CCD, with a 400 second total acquisition period for the synthetic samples and 20 seconds for FeS_2. Raman shifts were calibrated with a silicon chip at 521 cm^{-1}.

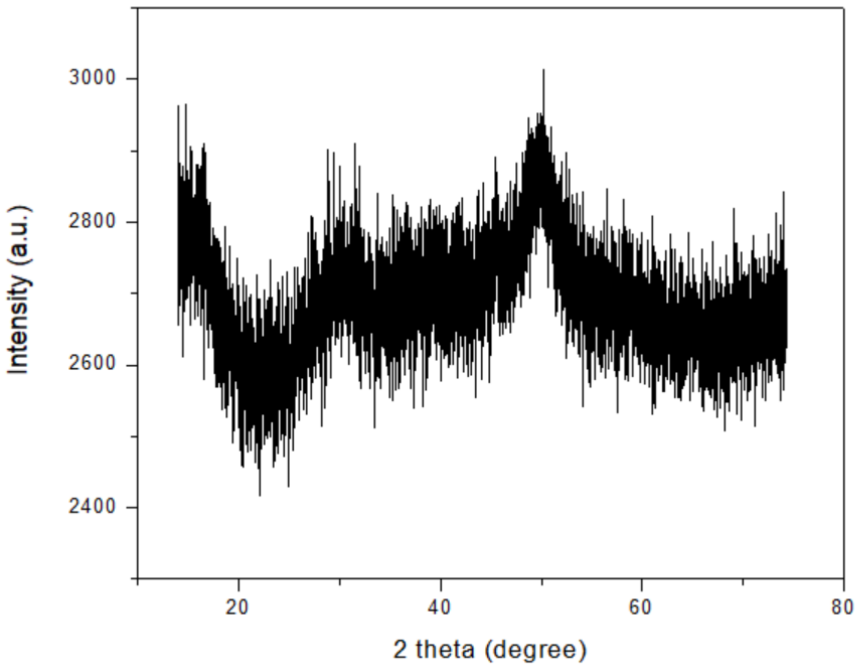

Figure S3. Attempted XRD analysis of powder of synthetic Ni-FeS. The lack of clear peaks may be the result of several FeS phases formed during the protocol used in our experiments.

SEM/EDX instrument. Secondary electron (SE) images obtained with TESCAN Vega-4 tungsten-filament SEM. X-ray spectra collected with Oxford Instruments Ultim Max energy dispersive spectrometer (EDS). Elemental concentrations are not standardized and therefore should be considered semiquantitative.

Conditions for SE imaging and EDS point analyses were:

15 mm working distance (this parameter varies from instrument to instrument and is not normally reported)

20 keV accelerating voltage

1 nA beam current

Instrument location: Colby College, Department of Geology

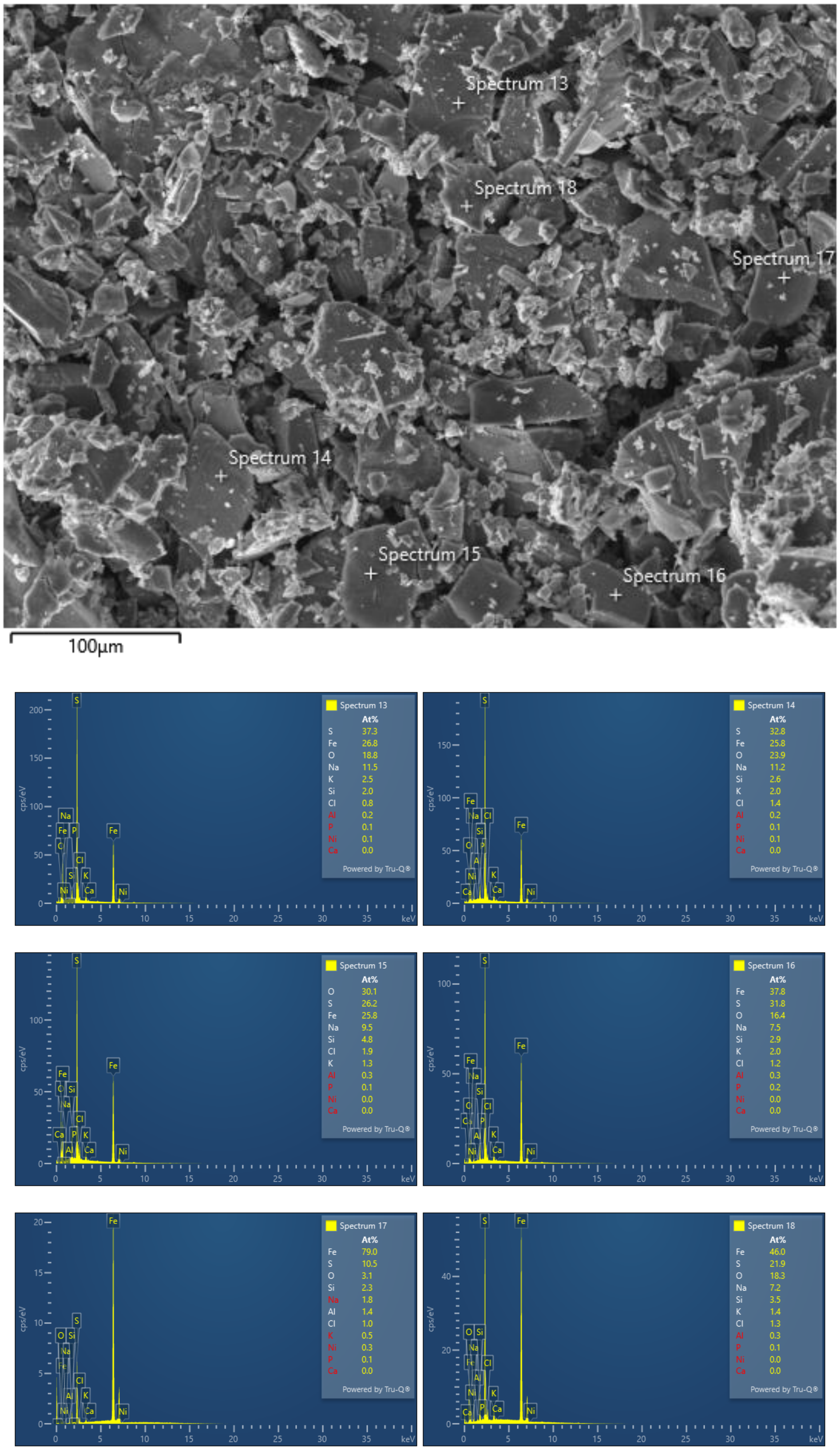

Figure S4. Scanning electron microscopy (SEM) images from of the FeS mineral and the points where Energy-dispersive x-ray spectroscopy (EDX) was performed.

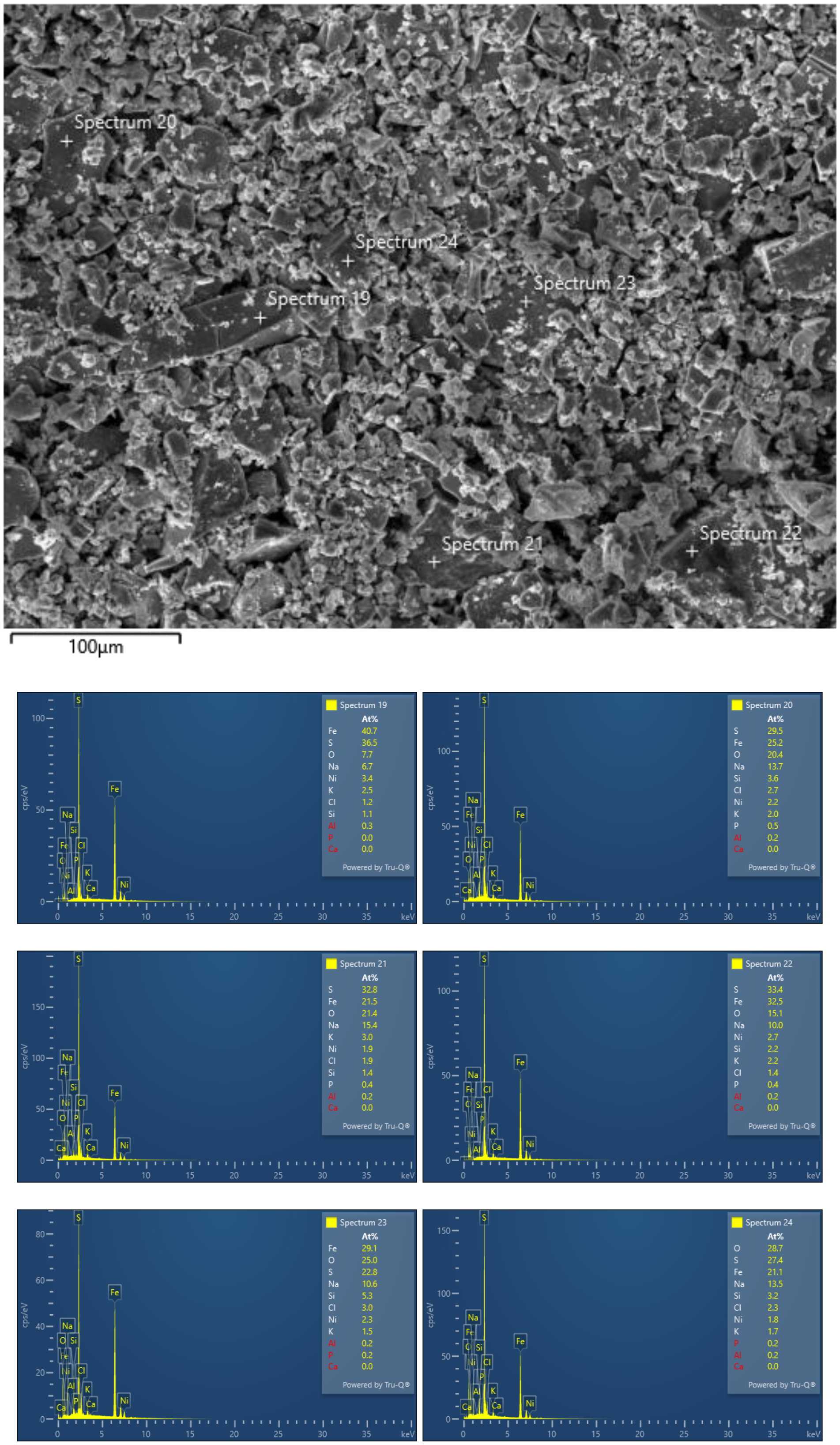

Figure S5. Scanning electron microscopy (SEM) images from of the Ni-FeS mineral and the points where Energy-dispersive x-ray spectroscopy (EDX) was performed.

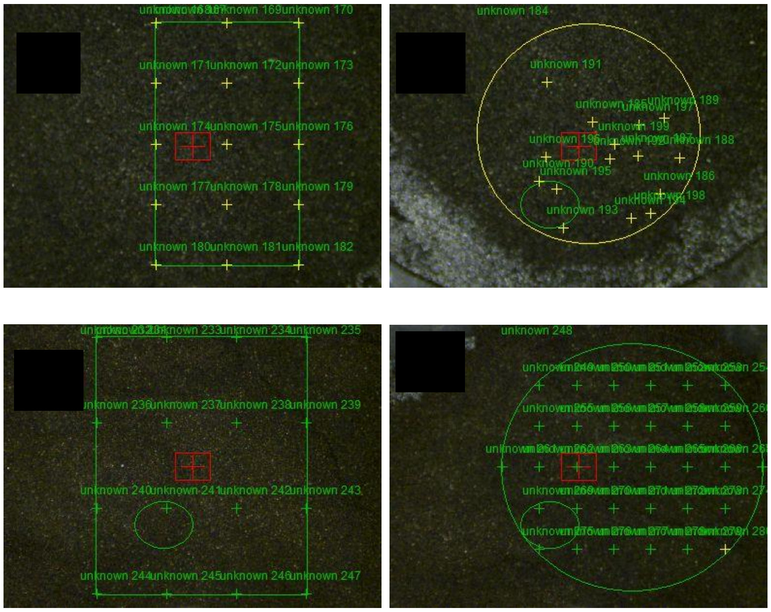

Figure S6. Images from of the minerals surfaces and the points where XRF analyzes were taken: a) FeS region 1; b) FeS region 2; c) Ni-FeS region 1; d) Ni-FeS region 2.

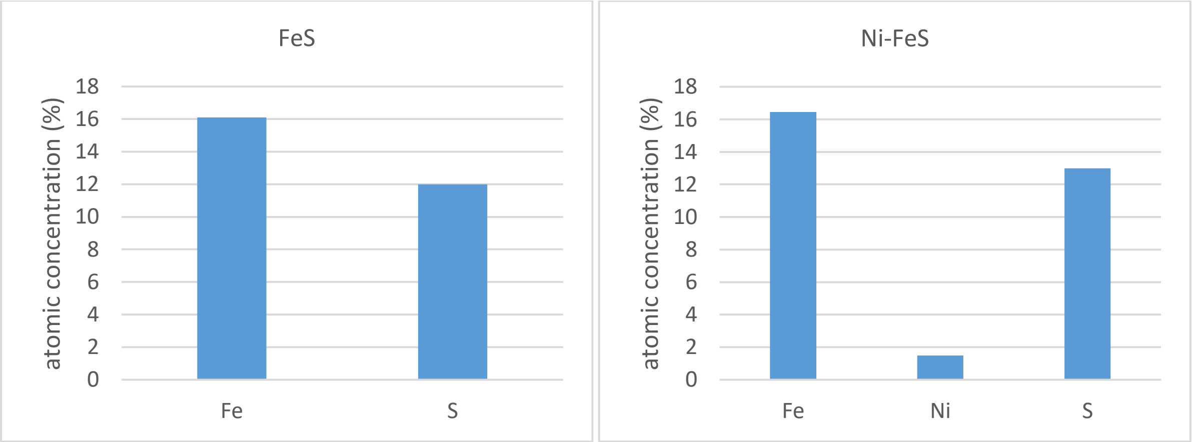

Figure S7. Average results from the XRF elemental analyses of the (a) FeS and (b) Ni-FeS synthesized by co-precipitation.

BET. Instrument: Micromeritics ASAP 2020
Analysis Adsorptive: N_2
Analysis Bath Temp: 77.300 K
Warm Free Space: 27.8223 cm^3 Measured
Equilibration Interval: 5 s
Sample Mass: 1.7010 g
Cold Free Space: 81.8066 cm^3
Ambient Temperature: 22.00 ˚C
Instrument Location: University of Maine, Orono

NMR analysis. NMR spectra were acquired on a Bruker 500 MHz NMR spectroscope. Formic acid and acetic acid were quantified using ^{1}H NMR. For sampling, 400 μL of sample were removed from the reactor with a syringe, and added to a microcentrifuge tube with 200 mg of chelant (Bio-rad Chelex 100 resin), to remove Fe ions from the sample, which was shaken for 20 minutes, and 200 μL of the supernatant was pipetted from the top and added to an NMR tube with 20 μL of D_2O. For quantification, DMSO was used as an internal standard (300 μL of an 1.4 mM concentration aqueous stock solution). Quantification was achieved by integration of the formyl peak or acetic acid methyl peak in comparison against the known concentration for the protons of DMSO. ^{1}H NMR spectra were conducted with water suppression for 256 scans. Water supression was performed with a Bruker noesygppr1d pulse program, with a relaxation delay d1 was set to 45 s. Fumarate and succinate were quantified similarly, although with a concentration of DMSO internal standard set to be a 1:1 ratio with the fumarate peak (1:1 integrations of DMSO and fumarate = 100% fumarate). Since succinate has 2x the protons as fumarate, a 2:1 ratio of succinate peak to the DMSO internal standard would indicate 100% conversion to succinate). The yields reported are based on relative integration of the succinate peak compared to the DMSO internal standard.

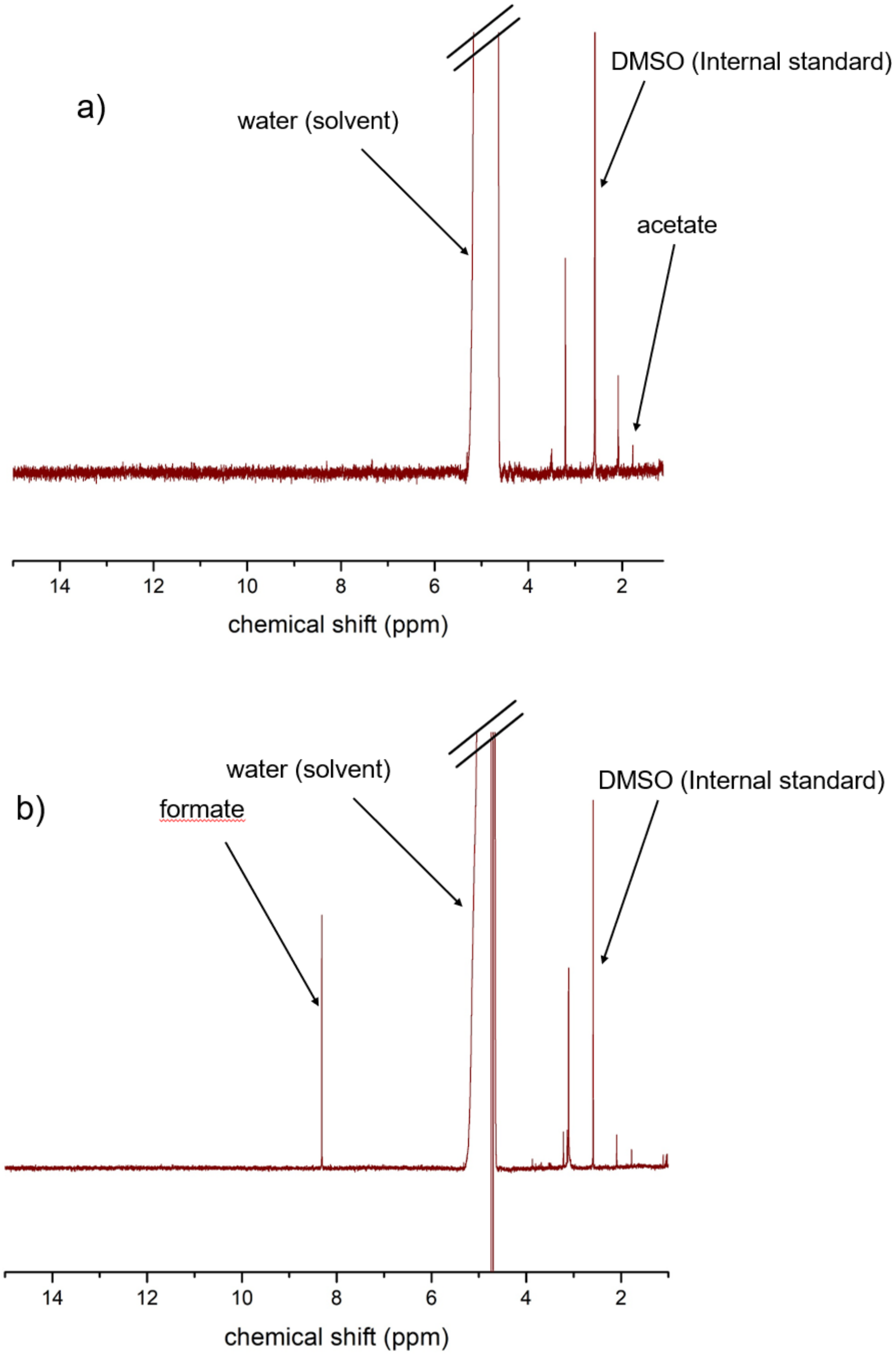

Figure S8. Reference spectra peaks of (a) formate, and (b) acetate with DMSO.

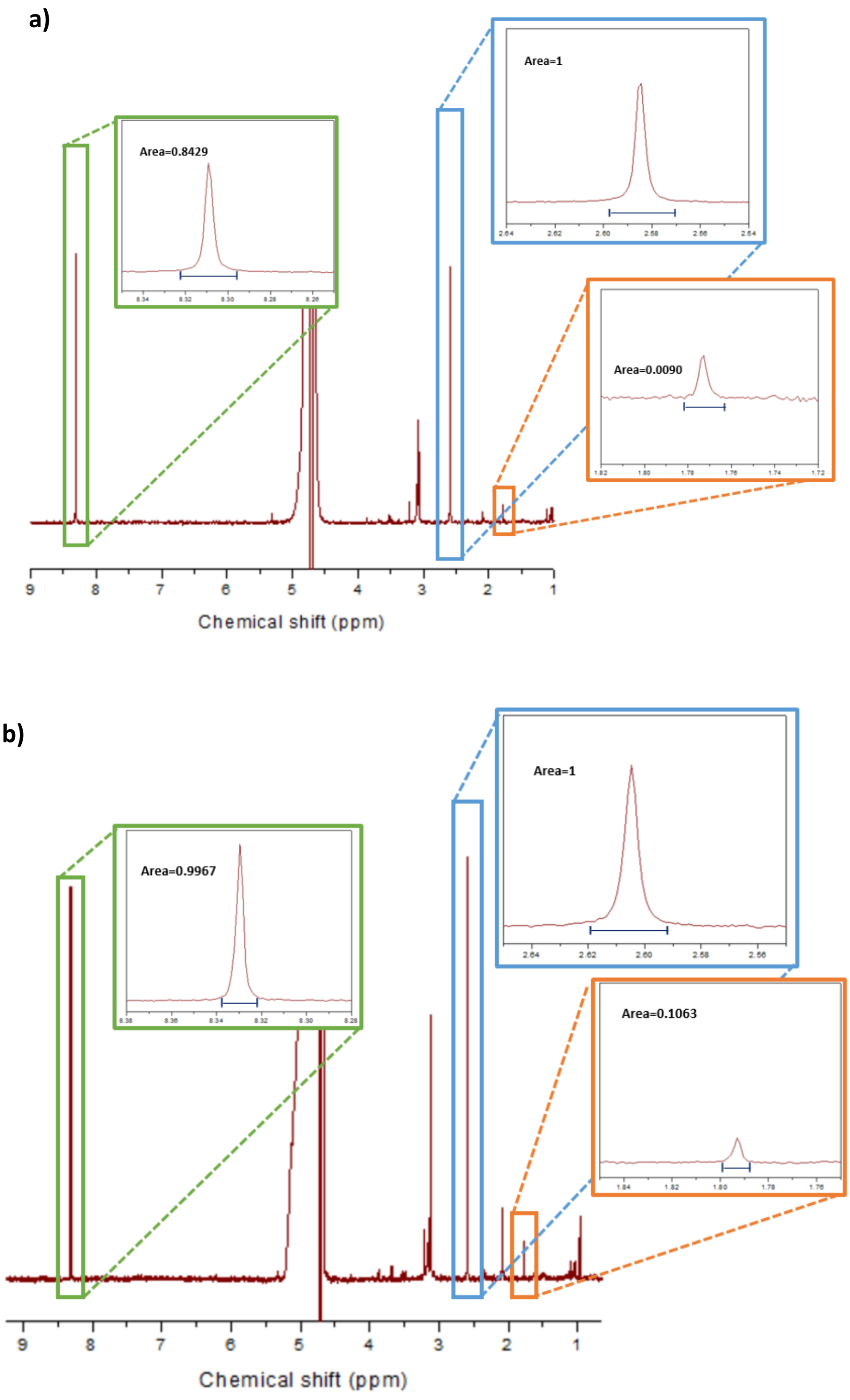

Figure S9. a) entry 1 of Table 2 with 54 μM DMSO as an internal standard Integration of the DMSO peak (1.00; 6 protons) relative to the formyl peak (0.8429; 1 proton), indicates a 815.8 μM concentration of formic acid; and to the acetyl peak (0.0090; 1 proton) indicates a 19.2 μM concentration of acetic acid. b) Entry 2 of Table 4. Integration of the DMSO peak (1.00; 6 protons) relative to the formyl peak (0.9967; 1 proton), indicates a 837.8 μM concentration of formic acid; and to the acetyl peak (0.1063; 1 proton) indicates a 29.8 μM concentration of acetic acid.

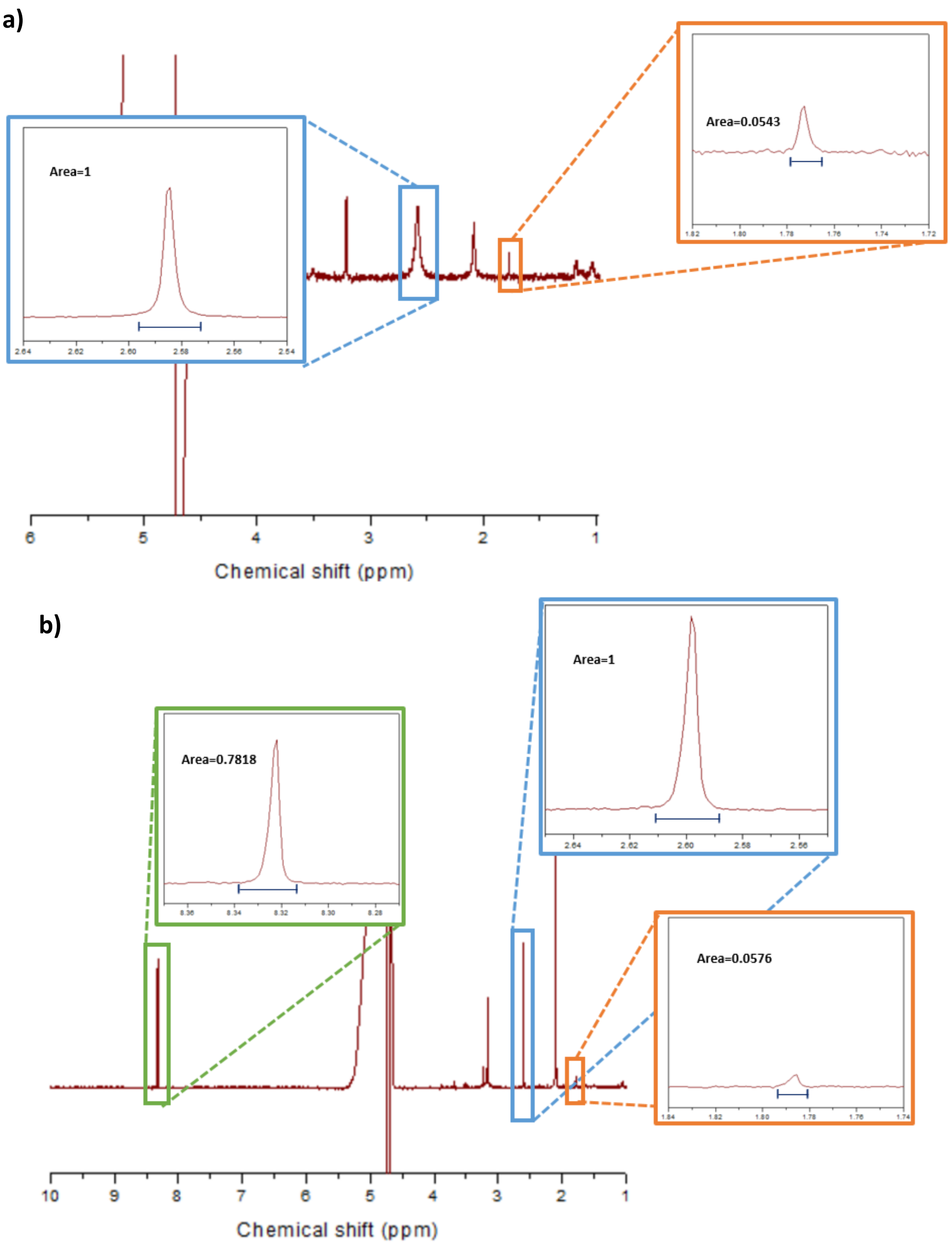

Figure S10. a) Entry 3 of Table 3. Integration of the DMSO peak (1.00; 6 protons) relative the acetyl peak (0.0543; 1 proton) indicates a 7.6 μM concentration of acetic acid; b) entry 1 of Table 4. Integration of the DMSO peak (1.00; 6 protons) relative to the formyl peak (0.7818; 1 proton), indicates a 678.4 μM concentration of formic acid; and to the acetyl peak (0.0576; 1 proton) indicates a 18.5 μM concentration of acetic acid.

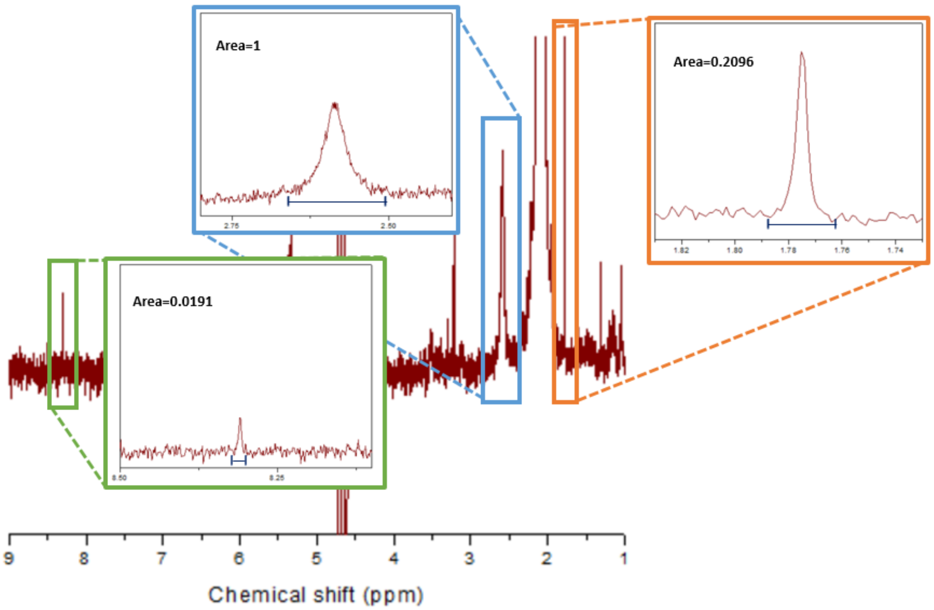

Figure S11. ^{1}H NMR spectra of Experiment for entry 4 of Table 4. Integration of the DMSO peak (1.00; 6 protons) relative to the formyl peak (0.0191; 1 proton), indicates a 16.1 μM concentration of formic acid; and to the acetyl peak (0.2096; 1 proton) indicates a 58.7 μM concentration of acetic acid.

Table S3. Names of all NMR and Chronoamperometry raw data files and their respective results shown in Tables in the Results section.

NMR File name	Chronoamperometry file name	Results (Table, entry)
NMR_0303	-	Table 1, entry 1
NMR_0228_OS	-	Table 1, entry 2
NMR_0305_OS	-	Table 1, entry 3
NMR_0305_VS	-	Table 1, entry 4
NMR_0228_VS	-	Table 1, entry 5
NMR_1226_18h	-	Table 2, entry 1
NMR_1101	-	Table 2, entry 2
NMR_128	-	Table 2, entry 3
NMR_0423	Manual Experiment(2024-04-23 21_54_21)	Table 3, entry 1
NMR_0429	Manual Experiment(2024-04-29 21_01_25)	Table 3, entry 2
NMR_0313		Table 3, entry 3
NMR_0416	Manual Experiment(2024-04-16 20_09_17)	Table 3, entry 4
NMR_0426	-	Table 3, entry 5
NMR_0422	-	Table 3, entry 6
NMR_0603	Manual Experiment(2024-06-03 21_54_54)	Table 4, entry 1
NMR_0618	Manual Experiment(2024-06-18 22_25_00)	Table 4, entry 2
NMR_0528	Manual Experiment(2024-05-28 20_56_24)	Table 4, entry 3
NMR_0530	Manual Experiment(2024-05-30 21_40_09)	Table 4, entry 4

EXPERIMENTAL RESULTS GRAPHS

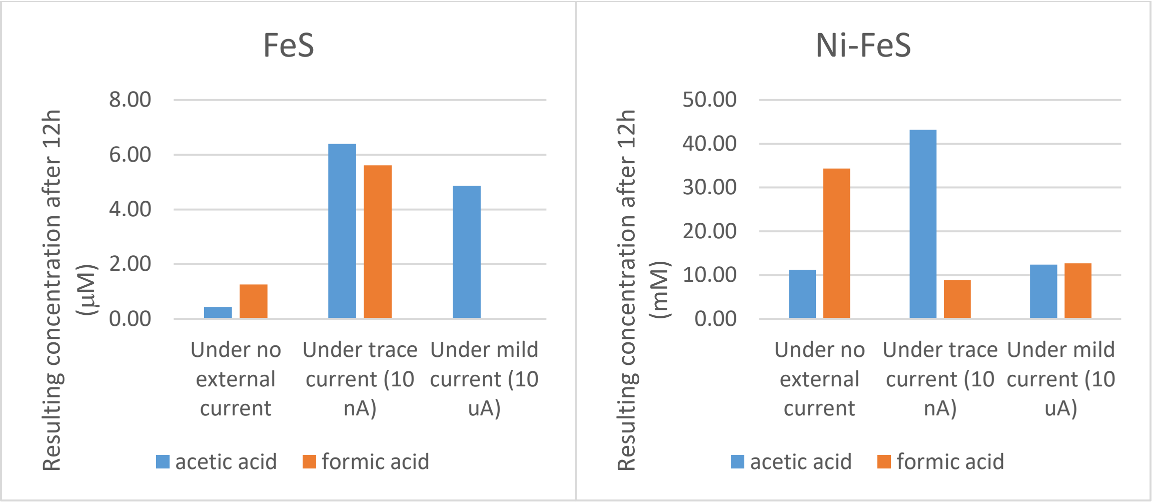

Figure S5. Production of different organics formed from electrochemically induced CO_2 reduction using different minerals under room temperature after 12 hours in the PTFE reactor (Fig. S2).

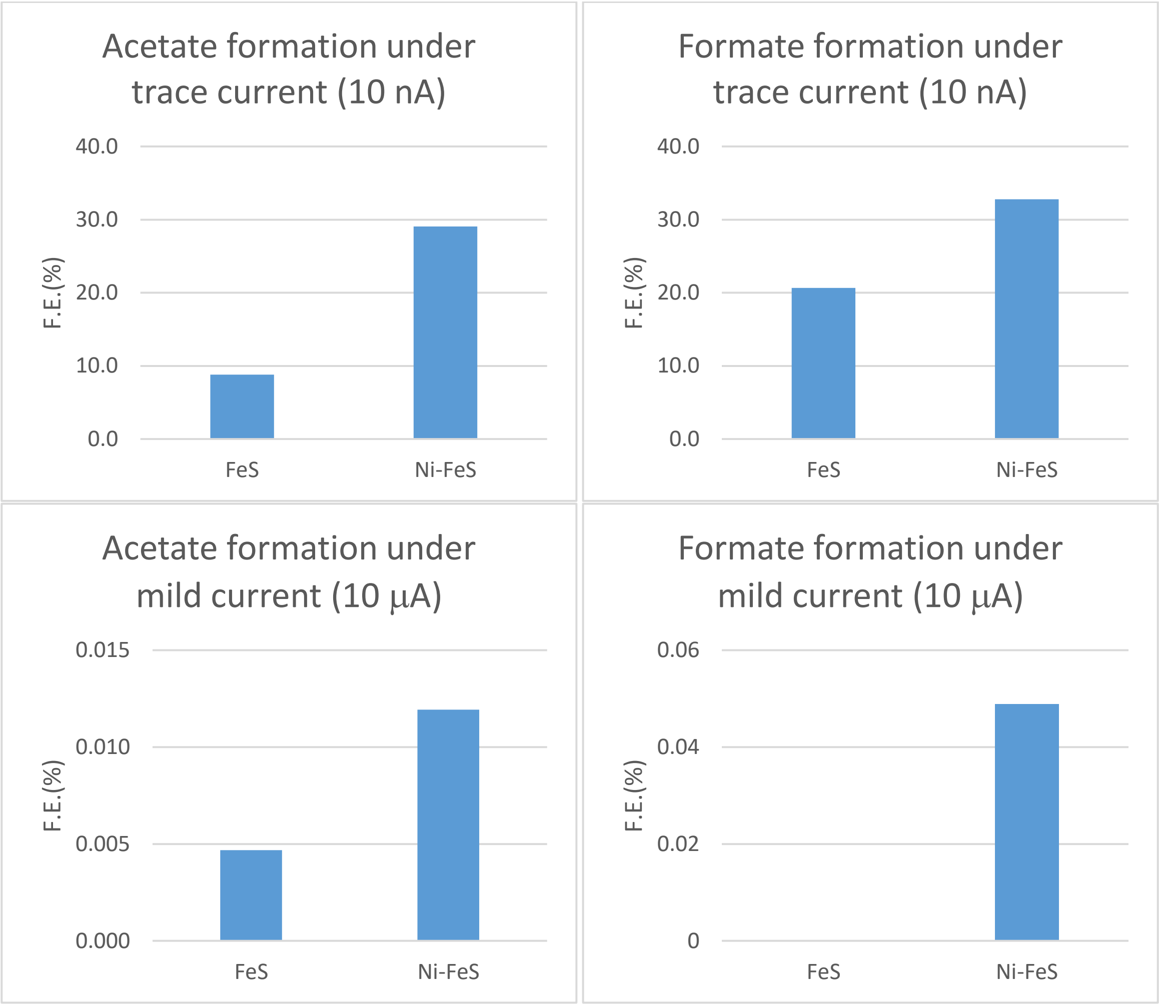

Figure S13. Current efficiencies from current-induced CO_2 reduction using different minerals under room temperature after 12 hours in the PTFE reactor (Fig. S2).

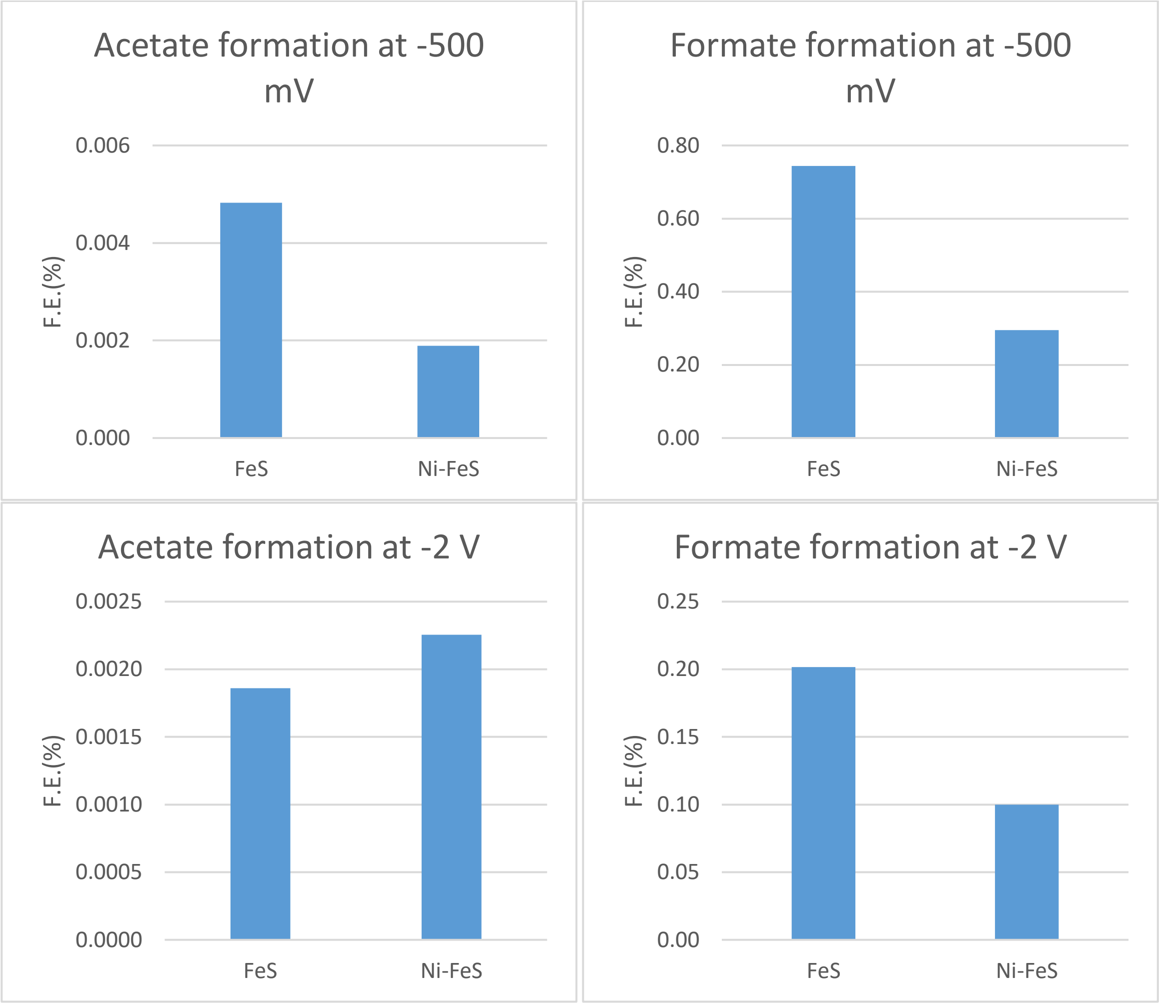

Figure S6. Current efficiencies from voltage-induced CO_2 reduction using different minerals under room temperature after 12 hours in the PTFE reactor (Fig. S2).

BET analysis report

Sample: 041725A
Operator: BGF
Submitter: Reuben Hudson, COA
File: C:\2020\HUDSON\041725A.SMP

Started:	4/18/2025 4:12:52PM	Analysis Adsorptive:	N2
Completed:	4/18/2025 10:15:32PM	Analysis Bath Temp.:	77.300 K
Report Time:	4/18/2025 10:50:08PM	Thermal Correction:	No
Sample Mass:	1.7010 g	Warm Free Space:	27.8223 cm³ Measured
Cold Free Space:	81.8066 cm³	Equilibration Interval:	5 s
Ambient Temperature:	22.00 °C	Low Pressure Dose:	None
Automatic Degas:	Yes		

Comments: Ni-FeS repeat degas after 041525B to repeat isotherm (.IIII)

Summary Report

Surface Area

Single point surface area at P/Po = 0.211118584: 6.2687 m²/g

BET Surface Area: 6.5832 m²/g

t-Plot External Surface Area: 7.2148 m²/g

BJH Adsorption cumulative surface area of pores between 17.000 Å and 3000.000 Å diameter: 6.470 m²/g

BJH Desorption cumulative surface area of pores between 17.000 Å and 3000.000 Å diameter: 7.3000 m²/g

Pore Volume

Single point adsorption total pore volume of pores less than 3619.008 Å diameter at P/Po = 0.994689451: 0.009924 cm³/g

t-Plot micropore volume: -0.000377 cm³/g

BJH Adsorption cumulative volume of pores between 17.000 Å and 3000.000 Å diameter: 0.010317 cm³/g

BJH Desorption cumulative volume of pores between 17.000 Å and 3000.000 Å diameter: 0.010815 cm³/g

Pore Size

Adsorption average pore width (4V/A by BET): 60.3015 Å

BJH Adsorption average pore diameter (4V/A): 63.786 Å

BJH Desorption average pore diameter (4V/A): 59.260 Å

Alpha-S

Slope: 2.4171 ± 0.0706 cm³/g STP

Y-Intercept: -0.1330 ± 0.0519 cm³/g STP

Sample: 041725A
Operator: BGF
Submitter: Reuben Hudson, COA
File: C:\2020\HUDSON\041725A.SMP

Started: 4/18/2025 4:12:52PM
Completed: 4/18/2025 10:15:32PM
Report Time: 4/18/2025 10:50:09PM
Sample Mass: 1.7010 g
Cold Free Space: 81.8066 cm³
Ambient Temperature: 22.00 °C
Automatic Degas: Yes

Analysis Adsorptive: N2
Analysis Bath Temp.: 77.300 K
Thermal Correction: No
Warm Free Space: 27.8223 cm³ Measured
Equilibration Interval: 5 s
Low Pressure Dose: None

Comments: Ni-FeS repeat degas after 041525B to repeat isotherm (.IIII)

Isotherm Tabular Report

Relative Pressure (P/Po)	Absolute Pressure (mmHg)	Quantity Adsorbed (cm³/g STP)	Elapsed Time (h:min)	Saturation Pressure (mmHg)
			02:12	763.583923
0.011239479	8.581531	1.0187	02:33	
0.032428743	24.759579	1.1930	02:36	
0.056459808	43.107109	1.3088	02:38	
0.070025554	53.464119	1.3659	02:40	
0.090271983	68.921577	1.4433	02:42	
0.110708267	84.523727	1.5111	02:44	
0.130708442	99.793076	1.5765	02:45	
0.150712713	115.064941	1.6394	02:47	
0.170871557	130.455109	1.7018	02:48	
0.190996633	145.819366	1.7632	02:49	
0.211118584	161.180435	1.8254	02:51	
0.231163256	176.482986	1.8867	02:52	
0.251278429	191.839233	1.9482	02:53	
0.271533254	207.301102	2.0106	02:55	
0.291712527	222.705963	2.0738	02:56	
0.311943062	238.148834	2.1362	02:58	
0.332052782	253.500275	2.1999	02:59	
0.352016485	268.740112	2.2642	03:00	
0.372118386	284.084137	2.3296	03:02	
0.392198794	299.412750	2.3908	03:03	
0.412201739	314.682098	2.4566	03:04	
0.432345444	330.058777	2.5230	03:05	
0.452508961	345.449005	2.5883	03:07	
0.472585729	360.774261	2.6527	03:08	
0.492673083	376.105896	2.7208	03:10	
0.512925327	391.564789	2.7865	03:11	
0.532805681	406.739655	2.8538	03:12	
0.553100595	422.229095	2.9237	03:14	
0.573121566	437.510986	2.9928	03:15	
0.590919785	451.095947	3.0554	03:16	
0.608642278	464.622986	3.1178	03:17	
0.617296738	471.225647	3.1577	03:19	
0.626517443	478.262451	3.1943	03:20	
0.635122887	484.829529	3.2269	03:21	
0.644096160	491.677338	3.2627	03:22	
0.653050615	498.508636	3.2951	03:24	
0.661803315	505.187927	3.3291	03:25	
0.670759220	512.022278	3.3606	03:26	
0.679427992	518.637390	3.3943	03:27	
0.688562917	525.606079	3.4290	03:29	
0.697306474	532.278137	3.4648	03:30	
0.706269991	539.118042	3.4999	03:31	
0.715084140	545.843872	3.5369	03:32	
0.723945532	552.603394	3.5745	03:34	

Sample: 041725A
Operator: BGF
Submitter: Reuben Hudson, COA
File: C:\2020\HUDSON\041725A.SMP

Started: 4/18/2025 4:12:52PM
Completed: 4/18/2025 10:15:32PM
Report Time: 4/18/2025 10:50:09PM
Sample Mass: 1.7010 g
Cold Free Space: 81.8066 cm³
Ambient Temperature: 22.00 °C
Automatic Degas: Yes

Analysis Adsorptive: N2
Analysis Bath Temp.: 77.300 K
Thermal Correction: No
Warm Free Space: 27.8223 cm³ Measured
Equilibration Interval: 5 s
Low Pressure Dose: None

Comments: Ni-FeS repeat degas after 041525B to repeat isotherm (.IIII)

Isotherm Tabular Report

Relative Pressure (P/Po)	Absolute Pressure (mmHg)	Quantity Adsorbed (cm³/g STP)	Elapsed Time (h:min)	Saturation Pressure (mmHg)
0.732822954	559.377380	3.6092	03:35	
0.741636321	566.102417	3.6449	03:36	
0.750391072	572.782654	3.6800	03:37	
0.753818435	575.393982	3.7016	03:39	
0.757609949	578.285645	3.7192	03:40	
0.761010239	580.878662	3.7352	03:41	
0.764647647	583.652649	3.7526	03:42	
0.768236567	586.387146	3.7681	03:44	
0.771752026	589.067993	3.7860	03:45	
0.775413129	591.859985	3.8012	03:46	
0.778957915	594.563171	3.8181	03:47	
0.782575577	597.319458	3.8357	03:49	
0.786101166	600.007935	3.8524	03:50	
0.789682201	602.738708	3.8720	03:51	
0.793285575	605.486511	3.8899	03:52	
0.796876565	608.222290	3.9089	03:54	
0.800480975	610.970825	3.9255	03:55	
0.803709249	613.432251	3.9438	03:56	
0.812585664	620.204590	3.9825	03:57	
0.821378748	626.910645	4.0284	03:59	
0.830614568	633.957153	4.0779	04:00	
0.839364813	640.632996	4.1289	04:01	
0.848217492	647.386963	4.1828	04:02	
0.857122298	654.177917	4.2393	04:04	
0.865933098	660.899780	4.3009	04:05	
0.874961013	667.787292	4.3634	04:06	
0.894525729	682.713745	4.4921	04:08	
0.914718559	698.122253	4.6487	04:09	
0.935035595	713.625427	4.8441	04:10	
0.954977253	728.838928	5.1038	04:12	
			04:14	763.193909
0.974552487	743.772522	5.5123	04:16	
0.985526027	752.147461	5.8385	04:18	
0.990262768	755.762512	6.0542	04:20	
0.992788485	757.690125	6.2105	04:21	
0.994689451	759.140930	6.4161	04:23	
0.965961113	737.215637	5.9735	04:25	
0.946977124	722.727173	5.7349	04:26	
0.930143379	709.879761	5.5455	04:27	
0.913443348	697.134399	5.3922	04:29	
0.897667170	685.094116	5.2858	04:30	
0.881622441	672.848877	5.2051	04:31	
0.865481105	660.529907	5.1413	04:33	
0.849155350	648.070190	5.0880	04:34	
0.832815759	635.599915	5.0421	04:35	

Sample: 041725A
Operator: BGF
Submitter: Reuben Hudson, COA
File: C:\2020\HUDSON\041725A.SMP

Started: 4/18/2025 4:12:52PM
Completed: 4/18/2025 10:15:32PM
Report Time: 4/18/2025 10:50:09PM
Sample Mass: 1.7010 g
Cold Free Space: 81.8066 cm³
Ambient Temperature: 22.00 °C
Automatic Degas: Yes

Analysis Adsorptive: N2
Analysis Bath Temp.: 77.300 K
Thermal Correction: No
Warm Free Space: 27.8223 cm³ Measured
Equilibration Interval: 5 s
Low Pressure Dose: None

Comments: Ni-FeS repeat degas after 041525B to repeat isotherm (.IIII)

Isotherm Tabular Report

Relative Pressure (P/Po)	Absolute Pressure (mmHg)	Quantity Adsorbed (cm³/g STP)	Elapsed Time (h:min)	Saturation Pressure (mmHg)
0.816585412	623.213013	5.0045	04:36	
0.800485102	610.925354	4.9699	04:38	
0.784212529	598.506226	4.9387	04:39	
0.767998017	586.131409	4.9097	04:40	
0.751839726	573.799500	4.8822	04:42	
0.740117396	564.853088	4.8620	04:43	
0.734352279	560.453186	4.8488	04:44	
0.728592600	556.057434	4.8367	04:45	
0.722887143	551.703064	4.8263	04:47	
0.717234868	547.389282	4.8181	04:48	
0.711612823	543.098572	4.8077	04:49	
0.705794603	538.658142	4.7975	04:50	
0.700143768	534.345459	4.7894	04:52	
0.694376412	529.943848	4.7773	04:53	
0.688679272	525.595825	4.7667	04:54	
0.682965657	521.235229	4.7555	04:55	
0.677318341	516.925232	4.7470	04:57	
0.671674943	512.618225	4.7350	04:58	
0.665729246	508.080505	4.7224	04:59	
0.660153905	503.825439	4.7121	05:00	
0.654351361	499.396973	4.7027	05:02	
0.638420874	487.238922	4.6739	05:03	
0.621980797	474.691956	4.6420	05:04	
0.605698148	462.265137	4.6042	05:06	
0.589469640	449.879639	4.5674	05:07	
0.573403199	437.617828	4.5245	05:08	
0.557067967	425.150879	4.4790	05:09	
0.540941185	412.843018	4.4318	05:11	
0.524643701	400.404877	4.3813	05:12	
0.508521998	388.100891	4.3274	05:13	
0.492756256	376.068573	4.2253	05:15	
0.479632593	366.052673	3.7957	05:20	
0.459950477	351.031403	3.1036	05:25	
0.439918718	335.743286	2.7916	05:28	
0.410979090	313.656738	2.5777	05:31	
0.393052509	299.975281	2.4879	05:32	
0.378984041	289.238312	2.4293	05:34	
0.362527570	276.678833	2.3715	05:35	
0.345916790	264.001587	2.3158	05:36	
0.329598012	251.547195	2.2626	05:37	
0.313358369	239.153198	2.2106	05:39	
0.297080878	226.730316	2.1602	05:40	
0.280968951	214.433792	2.1064	05:41	
0.264651793	201.980637	2.0564	05:43	
0.248682619	189.793060	2.0022	05:44	

Sample: 041725A
Operator: BGF
Submitter: Reuben Hudson, COA
File: C:\2020\HUDSON\041725A.SMP

Started: 4/18/2025 4:12:52PM
Completed: 4/18/2025 10:15:32PM
Report Time: 4/18/2025 10:50:09PM
Sample Mass: 1.7010 g
Cold Free Space: 81.8066 cm³
Ambient Temperature: 22.00 °C
Automatic Degas: Yes

Analysis Adsorptive: N2
Analysis Bath Temp.: 77.300 K
Thermal Correction: No
Warm Free Space: 27.8223 cm³ Measured
Equilibration Interval: 5 s
Low Pressure Dose: None

Comments: Ni-FeS repeat degas after 041525B to repeat isotherm (.IIII)

Isotherm Tabular Report

Relative Pressure (P/Po)	Absolute Pressure (mmHg)	Quantity Adsorbed (cm³/g STP)	Elapsed Time (h:min)	Saturation Pressure (mmHg)
0.232142816	177.169983	1.9501	05:45	
0.216203632	165.005295	1.8986	05:47	
0.199948793	152.599701	1.8444	05:48	

Sample: 041725A
Operator: BGF
Submitter: Reuben Hudson, COA
File: C:\2020\HUDSON\041725A.SMP

Started:	4/18/2025 4:12:52PM	Analysis Adsorptive:	N2
Completed:	4/18/2025 10:15:32PM	Analysis Bath Temp.:	77.300 K
Report Time:	4/18/2025 10:50:09PM	Thermal Correction:	No
Sample Mass:	1.7010 g	Warm Free Space:	27.8223 cm³ Measured
Cold Free Space:	81.8066 cm³	Equilibration Interval:	5 s
Ambient Temperature:	22.00 °C	Low Pressure Dose:	None
Automatic Degas:	Yes		

Comments: Ni-FeS repeat degas after 041525B to repeat isotherm (.IIII)

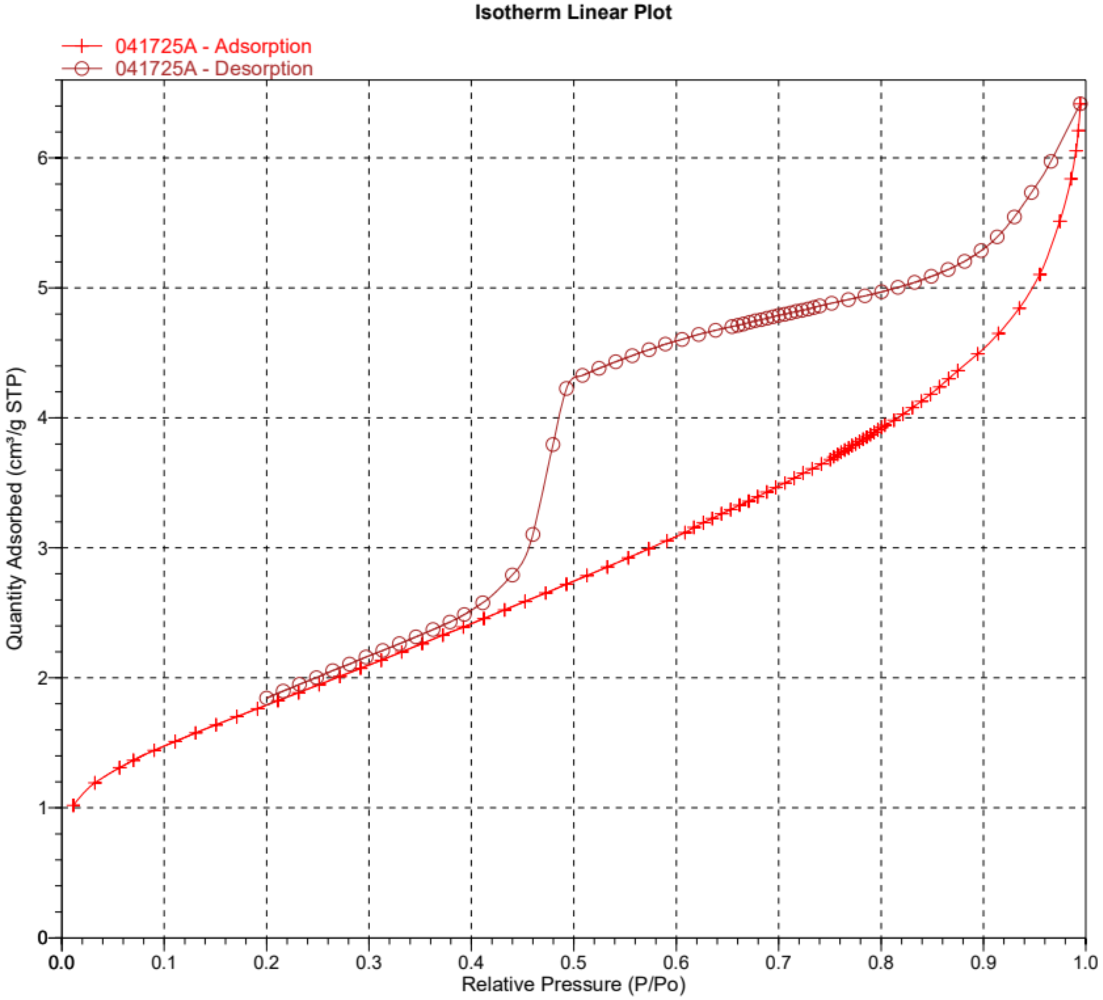

Sample: 041725A
Operator: BGF
Submitter: Reuben Hudson, COA
File: C:\2020\HUDSON\041725A.SMP

Started: 4/18/2025 4:12:52PM
Completed: 4/18/2025 10:15:32PM
Report Time: 4/18/2025 10:50:10PM
Sample Mass: 1.7010 g
Cold Free Space: 81.8066 cm³
Ambient Temperature: 22.00 °C
Automatic Degas: Yes

Analysis Adsorptive: N2
Analysis Bath Temp.: 77.300 K
Thermal Correction: No
Warm Free Space: 27.8223 cm³ Measured
Equilibration Interval: 5 s
Low Pressure Dose: None

Comments: Ni-FeS repeat degas after 041525B to repeat isotherm (.IIII)

BET Surface Area Report

BET Surface Area: 6.5832 ± 0.0416 m²/g
Slope: 0.651715 ± 0.004145 g/cm³ STP
Y-Intercept: 0.009548 ± 0.000554 g/cm³ STP
C: 69.256998
Qm: 1.5123 cm³/g STP
Correlation Coefficient: 0.9999191
Molecular Cross-Sectional Area: 0.1620 nm²

Relative Pressure (P/Po)	Quantity Adsorbed (cm³/g STP)	1/[Q(Po/P - 1)]
0.056459808	1.3088	0.045719
0.070025554	1.3659	0.055127
0.090271983	1.4433	0.068753
0.130708442	1.5765	0.095378
0.170871557	1.7018	0.121100
0.211118584	1.8254	0.146609

Sample: 041725A
Operator: BGF
Submitter: Reuben Hudson, COA
File: C:\2020\HUDSON\041725A.SMP

Started:	4/18/2025 4:12:52PM	Analysis Adsorptive:	N2
Completed:	4/18/2025 10:15:32PM	Analysis Bath Temp.:	77.300 K
Report Time:	4/18/2025 10:50:10PM	Thermal Correction:	No
Sample Mass:	1.7010 g	Warm Free Space:	27.8223 cm³ Measured
Cold Free Space:	81.8066 cm³	Equilibration Interval:	5 s
Ambient Temperature:	22.00 °C	Low Pressure Dose:	None
Automatic Degas:	Yes		

Comments: Ni-FeS repeat degas after 041525B to repeat isotherm (.IIII)

BET Surface Area Plot

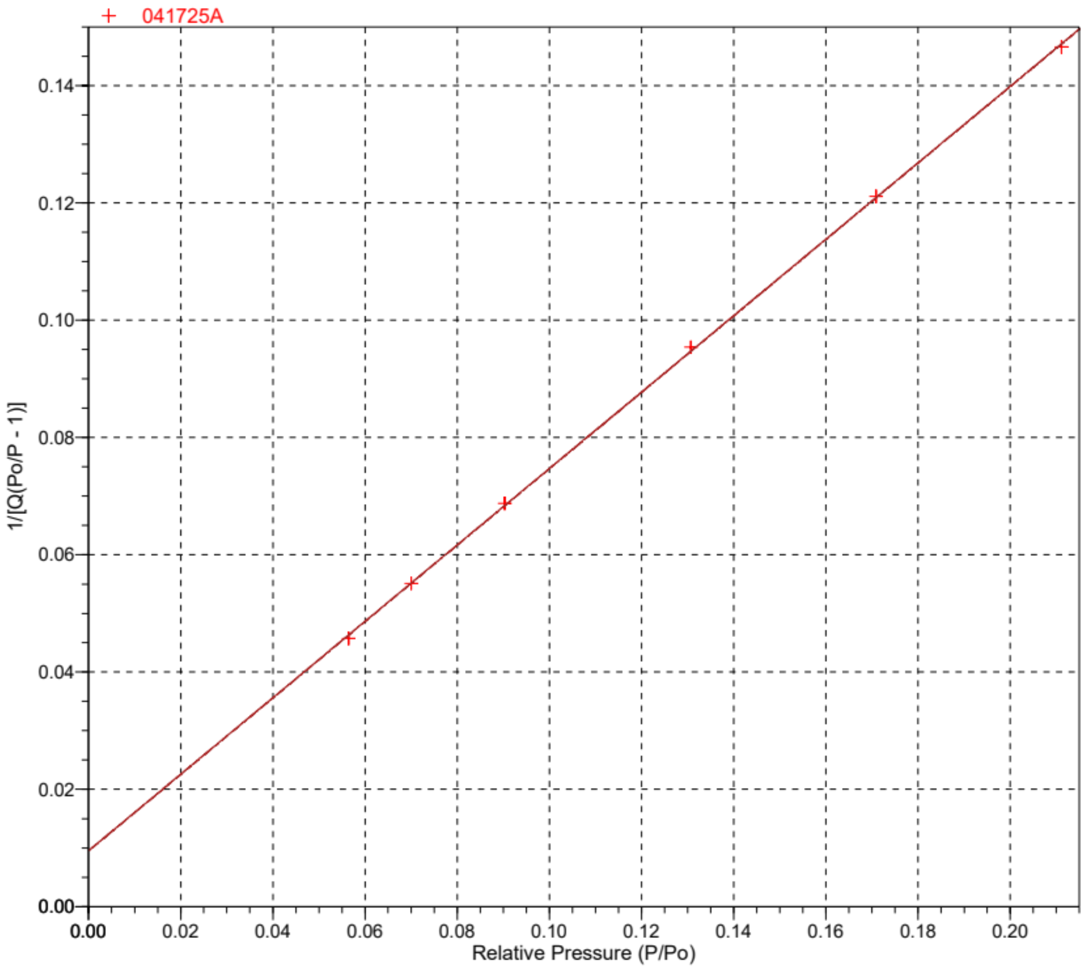

Sample: 041725A
Operator: BGF
Submitter: Reuben Hudson, COA
File: C:\2020\HUDSON\041725A.SMP

Started:	4/18/2025 4:12:52PM	Analysis Adsorptive:	N2
Completed:	4/18/2025 10:15:32PM	Analysis Bath Temp.:	77.300 K
Report Time:	4/18/2025 10:50:10PM	Thermal Correction:	No
Sample Mass:	1.7010 g	Warm Free Space:	27.8223 cm³ Measured
Cold Free Space:	81.8066 cm³	Equilibration Interval:	5 s
Ambient Temperature:	22.00 °C	Low Pressure Dose:	None
Automatic Degas:	Yes		

Comments: Ni-FeS repeat degas after 041525B to repeat isotherm (.IIII)

BJH Adsorption Pore Distribution Report

Kruk-Jaroniec-Sayari correction
Kruk-Jaroniec-Sayari
t = [60.65 / (0.03071 - log(P/Po))] ^ 0.3968

Diameter Range: 17.000 Å to 3000.000 Å
Adsorbate Property Factor: 9.53000 Å
Density Conversion Factor: 0.0015468
Fraction of Pores Open at Both Ends: 0.00

Pore Diameter Range (Å)	Average Diameter (Å)	Incremental Pore Volume (cm³/g)	Cumulative Pore Volume (cm³/g)	Incremental Pore Area (m²/g)	Cumulative Pore Area (m²/g)
2678.5 - 1992.5	2235.4	0.000250	0.000250	0.004	0.004
1992.5 - 1351.0	1550.7	0.000350	0.000600	0.009	0.014
1351.0 - 781.3	920.3	0.000545	0.001146	0.024	0.037
781.3 - 453.0	532.3	0.000715	0.001861	0.054	0.091
453.0 - 320.9	363.7	0.000471	0.002333	0.052	0.143
320.9 - 249.2	275.5	0.000365	0.002698	0.053	0.196
249.2 - 204.9	222.4	0.000301	0.002999	0.054	0.250
204.9 - 175.4	187.6	0.000252	0.003251	0.054	0.304
175.4 - 164.6	169.6	0.000125	0.003376	0.030	0.333
164.6 - 155.3	159.6	0.000125	0.003502	0.031	0.365
155.3 - 147.0	150.9	0.000116	0.003617	0.031	0.395
147.0 - 139.6	143.1	0.000111	0.003728	0.031	0.426
139.6 - 133.1	136.2	0.000106	0.003834	0.031	0.457
133.1 - 126.8	129.8	0.000103	0.003936	0.032	0.489
126.8 - 121.4	124.0	0.000096	0.004033	0.031	0.520
121.4 - 116.4	118.8	0.000080	0.004112	0.027	0.547
116.4 - 114.7	115.6	0.000040	0.004152	0.014	0.561
114.7 - 112.9	113.8	0.000035	0.004187	0.012	0.573
112.9 - 111.1	112.0	0.000041	0.004228	0.015	0.588
111.1 - 109.4	110.3	0.000038	0.004266	0.014	0.601
109.4 - 107.8	108.6	0.000043	0.004309	0.016	0.617
107.8 - 106.2	106.9	0.000036	0.004345	0.013	0.631
106.2 - 104.6	105.4	0.000038	0.004383	0.014	0.645
104.6 - 103.1	103.8	0.000037	0.004419	0.014	0.659
103.1 - 101.6	102.3	0.000032	0.004452	0.013	0.672
101.6 - 100.2	100.9	0.000040	0.004491	0.016	0.687
100.2 - 98.8	99.5	0.000033	0.004525	0.013	0.701
98.8 - 97.5	98.1	0.000038	0.004563	0.016	0.716
97.5 - 96.2	96.8	0.000035	0.004598	0.014	0.731
96.2 - 94.9	95.6	0.000039	0.004637	0.016	0.747
94.9 - 93.7	94.3	0.000050	0.004687	0.021	0.768
93.7 - 90.8	92.2	0.000076	0.004763	0.033	0.802
90.8 - 88.1	89.4	0.000078	0.004841	0.035	0.836
88.1 - 85.6	86.8	0.000076	0.004918	0.035	0.872
85.6 - 83.1	84.3	0.000085	0.005003	0.040	0.912
83.1 - 80.9	82.0	0.000084	0.005087	0.041	0.953
80.9 - 78.7	79.7	0.000079	0.005166	0.040	0.993

Sample: 041725A
Operator: BGF
Submitter: Reuben Hudson, COA
File: C:\2020\HUDSON\041725A.SMP

Started: 4/18/2025 4:12:52PM
Completed: 4/18/2025 10:15:32PM
Report Time: 4/18/2025 10:50:10PM
Sample Mass: 1.7010 g
Cold Free Space: 81.8066 cm³
Ambient Temperature: 22.00 °C
Automatic Degas: Yes

Analysis Adsorptive: N2
Analysis Bath Temp.: 77.300 K
Thermal Correction: No
Warm Free Space: 27.8223 cm³ Measured
Equilibration Interval: 5 s
Low Pressure Dose: None

Comments: Ni-FeS repeat degas after 041525B to repeat isotherm (.IIII)

Pore Diameter Range (Å)	Average Diameter (Å)	Incremental Pore Volume (cm³/g)	Cumulative Pore Volume (cm³/g)	Incremental Pore Area (m²/g)	Cumulative Pore Area (m²/g)
78.7 - 76.7	77.7	0.000082	0.005248	0.042	1.035
76.7 - 74.7	75.6	0.000079	0.005327	0.042	1.077
74.7 - 72.9	73.8	0.000078	0.005405	0.042	1.119
72.9 - 71.1	72.0	0.000071	0.005477	0.040	1.159
71.1 - 69.5	70.3	0.000079	0.005556	0.045	1.204
69.5 - 67.9	68.6	0.000075	0.005631	0.044	1.248
67.9 - 66.3	67.1	0.000086	0.005717	0.051	1.299
66.3 - 64.9	65.6	0.000077	0.005794	0.047	1.346
64.9 - 63.5	64.2	0.000088	0.005882	0.055	1.401
63.5 - 62.2	62.8	0.000100	0.005983	0.064	1.465
62.2 - 59.7	60.8	0.000148	0.006131	0.097	1.562
59.7 - 57.3	58.4	0.000151	0.006281	0.103	1.665
57.3 - 54.9	56.0	0.000167	0.006449	0.119	1.785
54.9 - 52.6	53.7	0.000171	0.006619	0.127	1.912
52.6 - 50.6	51.5	0.000165	0.006784	0.128	2.040
50.6 - 48.6	49.5	0.000160	0.006944	0.129	2.169
48.6 - 46.8	47.7	0.000170	0.007114	0.142	2.312
46.8 - 45.1	45.9	0.000158	0.007272	0.138	2.450
45.1 - 43.5	44.2	0.000162	0.007434	0.146	2.596
43.5 - 42.0	42.7	0.000167	0.007601	0.156	2.752
42.0 - 40.5	41.2	0.000165	0.007766	0.160	2.912
40.5 - 39.2	39.8	0.000148	0.007914	0.149	3.061
39.2 - 37.9	38.5	0.000164	0.008077	0.170	3.231
37.9 - 36.6	37.2	0.000159	0.008237	0.171	3.402
36.6 - 35.4	36.0	0.000156	0.008393	0.173	3.575
35.4 - 34.3	34.8	0.000148	0.008541	0.170	3.746
34.3 - 33.1	33.7	0.000151	0.008691	0.179	3.925
33.1 - 32.0	32.6	0.000144	0.008836	0.177	4.102
32.0 - 31.0	31.5	0.000139	0.008975	0.176	4.279
31.0 - 30.0	30.5	0.000135	0.009110	0.177	4.455
30.0 - 28.9	29.4	0.000133	0.009243	0.181	4.636
28.9 - 27.9	28.4	0.000124	0.009367	0.175	4.811
27.9 - 26.9	27.4	0.000120	0.009487	0.175	4.986
26.9 - 25.9	26.4	0.000114	0.009600	0.172	5.158
25.9 - 24.9	25.4	0.000111	0.009712	0.176	5.334
24.9 - 23.8	24.3	0.000099	0.009811	0.163	5.496
23.8 - 22.7	23.2	0.000116	0.009927	0.200	5.697
22.7 - 21.9	22.3	0.000077	0.010004	0.138	5.835
21.9 - 20.2	21.0	0.000120	0.010124	0.228	6.063
20.2 - 18.0	19.0	0.000193	0.010317	0.406	6.470

Sample: 041725A
Operator: BGF
Submitter: Reuben Hudson, COA
File: C:\2020\HUDSON\041725A.SMP

Started:	4/18/2025 4:12:52PM	Analysis Adsorptive:	N2
Completed:	4/18/2025 10:15:32PM	Analysis Bath Temp.:	77.300 K
Report Time:	4/18/2025 10:50:10PM	Thermal Correction:	No
Sample Mass:	1.7010 g	Warm Free Space:	27.8223 cm³ Measured
Cold Free Space:	81.8066 cm³	Equilibration Interval:	5 s
Ambient Temperature:	22.00 °C	Low Pressure Dose:	None
Automatic Degas:	Yes		

Comments: Ni-FeS repeat degas after 041525B to repeat isotherm (.IIII)

BJH Adsorption Cumulative Pore Volume (Larger)

Kruk-Jaroniec-Sayari : Kruk-Jaroniec-Sayari correction

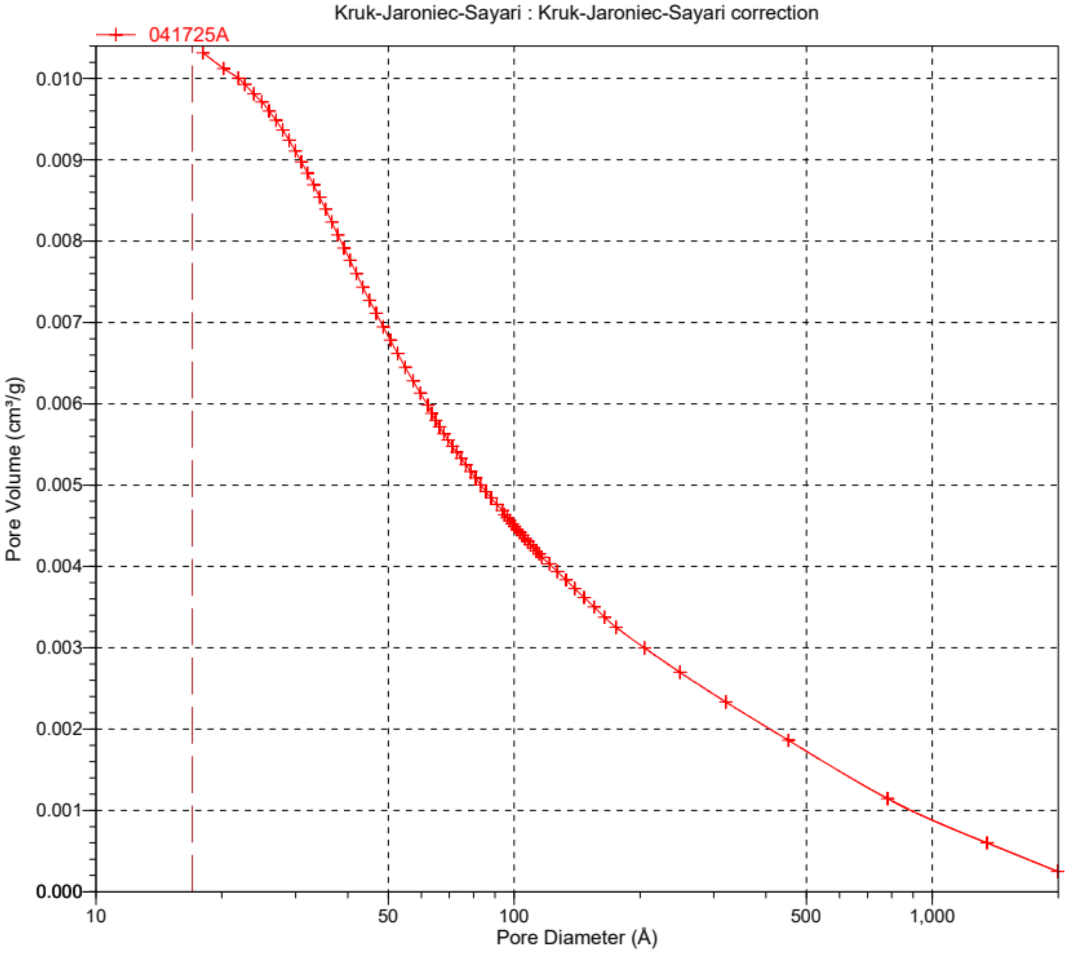

Sample: 041725A
Operator: BGF
Submitter: Reuben Hudson, COA
File: C:\2020\HUDSON\041725A.SMP

Started:	4/18/2025 4:12:52PM	Analysis Adsorptive:	N2
Completed:	4/18/2025 10:15:32PM	Analysis Bath Temp.:	77.300 K
Report Time:	4/18/2025 10:50:10PM	Thermal Correction:	No
Sample Mass:	1.7010 g	Warm Free Space:	27.8223 cm³ Measured
Cold Free Space:	81.8066 cm³	Equilibration Interval:	5 s
Ambient Temperature:	22.00 °C	Low Pressure Dose:	None
Automatic Degas:	Yes		

Comments: Ni-FeS repeat degas after 041525B to repeat isotherm (.IIII)

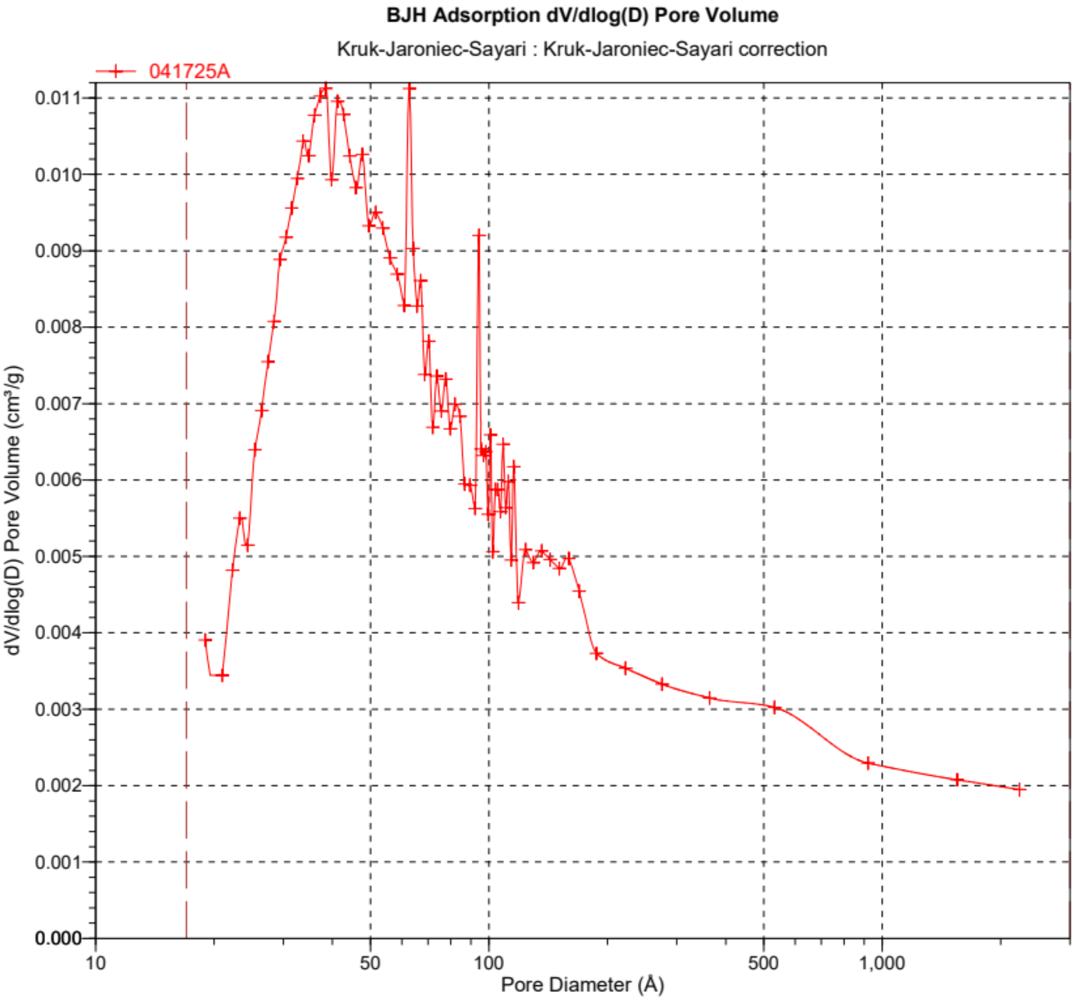

Sample: 041725A
Operator: BGF
Submitter: Reuben Hudson, COA
File: C:\2020\HUDSON\041725A.SMP

Started:	4/18/2025 4:12:52PM	Analysis Adsorptive:	N2
Completed:	4/18/2025 10:15:32PM	Analysis Bath Temp.:	77.300 K
Report Time:	4/18/2025 10:50:11PM	Thermal Correction:	No
Sample Mass:	1.7010 g	Warm Free Space:	27.8223 cm³ Measured
Cold Free Space:	81.8066 cm³	Equilibration Interval:	5 s
Ambient Temperature:	22.00 °C	Low Pressure Dose:	None
Automatic Degas:	Yes		

Comments: Ni-FeS repeat degas after 041525B to repeat isotherm (.IIII)

BJH Desorption Pore Distribution Report

Kruk-Jaroniec-Sayari correction
Kruk-Jaroniec-Sayari
t = [60.65 / (0.03071 - log(P/Po))] ^ 0.3968

Diameter Range: 17.000 Å to 3000.000 Å
Adsorbate Property Factor: 9.53000 Å
Density Conversion Factor: 0.0015468
Fraction of Pores Open at Both Ends: 0.00

Pore Diameter Range (Å)	Average Diameter (Å)	Incremental Pore Volume (cm³/g)	Cumulative Pore Volume (cm³/g)	Incremental Pore Area (m²/g)	Cumulative Pore Area (m²/g)
3625.0 - 591.0	662.8	0.000763	0.000763	0.046	0.046
591.0 - 388.2	447.3	0.000425	0.001188	0.038	0.084
388.2 - 299.9	332.1	0.000349	0.001538	0.042	0.126
299.9 - 245.8	267.1	0.000290	0.001828	0.043	0.170
245.8 - 210.7	225.3	0.000204	0.002032	0.036	0.206
210.7 - 184.3	195.6	0.000156	0.002187	0.032	0.238
184.3 - 164.0	172.9	0.000123	0.002311	0.029	0.266
164.0 - 147.8	155.0	0.000103	0.002414	0.027	0.293
147.8 - 134.7	140.5	0.000089	0.002503	0.025	0.318
134.7 - 123.8	128.7	0.000073	0.002576	0.023	0.341
123.8 - 114.7	118.9	0.000067	0.002643	0.023	0.363
114.7 - 106.9	110.5	0.000061	0.002704	0.022	0.385
106.9 - 100.1	103.3	0.000057	0.002761	0.022	0.407
100.1 - 94.2	97.0	0.000055	0.002815	0.023	0.430
94.2 - 90.4	92.2	0.000041	0.002856	0.018	0.448
90.4 - 88.6	89.5	0.000029	0.002885	0.013	0.461
88.6 - 86.9	87.7	0.000026	0.002912	0.012	0.473
86.9 - 85.3	86.0	0.000022	0.002933	0.010	0.483
85.3 - 83.7	84.5	0.000016	0.002950	0.008	0.491
83.7 - 82.2	83.0	0.000022	0.002972	0.011	0.501
82.2 - 80.7	81.5	0.000022	0.002994	0.011	0.512
80.7 - 79.4	80.0	0.000016	0.003010	0.008	0.520
79.4 - 78.0	78.7	0.000027	0.003037	0.014	0.534
78.0 - 76.7	77.3	0.000023	0.003061	0.012	0.546
76.7 - 75.4	76.1	0.000025	0.003086	0.013	0.559
75.4 - 74.2	74.8	0.000018	0.003103	0.009	0.569
74.2 - 73.1	73.6	0.000028	0.003131	0.015	0.584
73.1 - 71.9	72.5	0.000029	0.003161	0.016	0.600
71.9 - 70.8	71.3	0.000023	0.003184	0.013	0.613
70.8 - 69.7	70.2	0.000021	0.003205	0.012	0.625
69.7 - 66.9	68.2	0.000066	0.003271	0.039	0.664
66.9 - 64.2	65.5	0.000075	0.003346	0.046	0.710
64.2 - 61.7	62.9	0.000094	0.003440	0.060	0.770
61.7 - 59.5	60.5	0.000092	0.003533	0.061	0.831
59.5 - 57.4	58.4	0.000112	0.003645	0.077	0.907
57.4 - 55.4	56.3	0.000121	0.003765	0.086	0.993
55.4 - 53.5	54.4	0.000127	0.003892	0.093	1.086

Sample: 041725A
Operator: BGF
Submitter: Reuben Hudson, COA
File: C:\2020\HUDSON\041725A.SMP

Started: 4/18/2025 4:12:52PM
Completed: 4/18/2025 10:15:32PM
Report Time: 4/18/2025 10:50:11PM
Sample Mass: 1.7010 g
Cold Free Space: 81.8066 cm³
Ambient Temperature: 22.00 °C
Automatic Degas: Yes

Analysis Adsorptive: N2
Analysis Bath Temp.: 77.300 K
Thermal Correction: No
Warm Free Space: 27.8223 cm³ Measured
Equilibration Interval: 5 s
Low Pressure Dose: None

Comments: Ni-FeS repeat degas after 041525B to repeat isotherm (.IIII)

Pore Diameter Range (Å)	Average Diameter (Å)	Incremental Pore Volume (cm³/g)	Cumulative Pore Volume (cm³/g)	Incremental Pore Area (m²/g)	Cumulative Pore Area (m²/g)
53.5 - 51.8	52.6	0.000138	0.004031	0.105	1.192
51.8 - 50.1	50.9	0.000150	0.004181	0.118	1.310
50.1 - 48.6	49.3	0.000313	0.004493	0.253	1.563
48.6 - 47.4	48.0	0.001428	0.005921	1.190	2.753
47.4 - 45.7	46.5	0.002309	0.008230	1.985	4.738
45.7 - 44.1	44.8	0.000973	0.009203	0.867	5.606
44.1 - 41.9	42.9	0.000565	0.009767	0.526	6.132
41.9 - 40.6	41.2	0.000193	0.009960	0.187	6.319
40.6 - 39.6	40.1	0.000108	0.010068	0.107	6.427
39.6 - 38.5	39.1	0.000086	0.010154	0.089	6.515
38.5 - 37.5	38.0	0.000078	0.010232	0.082	6.598
37.5 - 36.5	37.0	0.000071	0.010303	0.076	6.674
36.5 - 35.5	36.0	0.000067	0.010369	0.074	6.748
35.5 - 34.6	35.0	0.000060	0.010429	0.069	6.817
34.6 - 33.7	34.1	0.000074	0.010503	0.086	6.903
33.7 - 32.8	33.2	0.000056	0.010559	0.067	6.970
32.8 - 31.9	32.3	0.000075	0.010634	0.092	7.063
31.9 - 31.0	31.5	0.000059	0.010692	0.075	7.137
31.0 - 30.2	30.6	0.000059	0.010751	0.077	7.214
30.2 - 29.4	29.8	0.000064	0.010815	0.086	7.300

SI References

(1) Herschy, B.; Whicher, A.; Camprubi, E.; Watson, C.; Dartnell, L.; Ward, J.; Evans, J. R. G.; Lane, N. An Origin-of-Life Reactor to Simulate Alkaline Hydrothermal Vents. *J. Mol. Evol.* **2014**, *79* (5–6), 213–227. https://doi.org/10.1007/s00239-014-9658-4.

(2) Sojo, V.; Ohno, A.; McGlynn, S. E.; Yamada, Y. M. A.; Nakamura, R. Microfluidic Reactors for Carbon Fixation under Ambient-Pressure Alkaline-Hydrothermal-Vent Conditions. *Life* **2019**, *9* (1), 16. https://doi.org/10.3390/life9010016.

(3) Hudson, R.; de Graaf, R.; Strandoo Rodin, M.; Ohno, A.; Lane, N.; McGlynn, S. E.; Yamada, Y. M. A.; Nakamura, R.; Barge, L. M.; Braun, D.; Sojo, V. CO 2 Reduction Driven by a PH Gradient. *Proc. Natl. Acad. Sci.* **2020**, *117* (37), 22873–22879. https://doi.org/10.1073/pnas.2002659117.
